# The Role of P62/Nrf2/Keap1 Signaling Pathway in Lead‐Induced Neurological Dysfunction

**DOI:** 10.1111/cns.70566

**Published:** 2025-09-05

**Authors:** Dongjie Peng, Peiqi Wei, Zhenning Li, Ruokun Wei, Huishuai Li, Shaojun Li

**Affiliations:** ^1^ School of Public Health Guangxi Medical University Nanning Guangxi China; ^2^ Guangxi Colleges and Universities Key Laboratory of Prevention and Control of Highly Prevalent Diseases Guangxi Medical University Nanning China

**Keywords:** autophagy, lead, neurological dysfunction, oxidative stress, P62/Nrf2/Keap1

## Abstract

**Background:**

Lead (Pb) exposure is recognized for its contribution to the development of neurodegenerative diseases. However, the precise mechanisms underlying Pb‐induced neurological dysfunction remain elusive. This study aimed to investigate the role of oxidative stress and the autophagy‐related P62/kelch like ECH‐associated protein 1 (Keap1)/Nuclear factor erythroid 2‐related factor 2 (Nrf2) pathway in neuronal impairment caused by Pb.

**Methods:**

By employing both in vivo and in vitro approaches, we explored the involvement of the P62/Nrf2/Keap1 pathway in Pb‐induced neurotoxicity.

**Results:**

Our findings demonstrated that Pb exposure triggers excessive production of reactive oxygen species (ROS), upregulates Keap1 protein expressions, promotes Nrf2 degradation, and inhibits expression of antioxidant proteins such as heme Oxygenase‐1 (HO‐1) and glutathione peroxidase (GPx), resulting in oxidative damage in neurons. Furthermore, we observed that the autophagy protein P62 disrupts the normal autophagy process by interacting with the Nrf2/Keap1 axis, leading to an accumulation of Tau, a protein associated with Alzheimer's disease (AD), ultimately resulting in neurodegeneration. However, treatment with the antioxidant N‐acetylcysteine, Nrf2 activator Artemisitene, and autophagy activator Rapamycin attenuated these detrimental changes.

**Conclusion:**

The P62/Nrf2/Keap1 pathway mediates Pb‐induced neuronal dysfunction and highlights its potential as a therapeutic target for mitigating the neurodegenerative effects associated with Pb exposure.

## Graphical abstract

Pb exposure results in a significant increase in ROS, upregulation of Keap1 expression, degradation of Nrf2, and inhibition of downstream antioxidant protein HO‐1 and GPx expression, resulting in oxidative damage in neurons. Simultaneously, the P62 protein competitively binds with Keap1, preventing P62 from exerting its autophagy function and resulting in the accumulation of damaged proteins within cells, ultimately causing neurodegeneration. However, the antioxidant NAC can mitigate these changes.

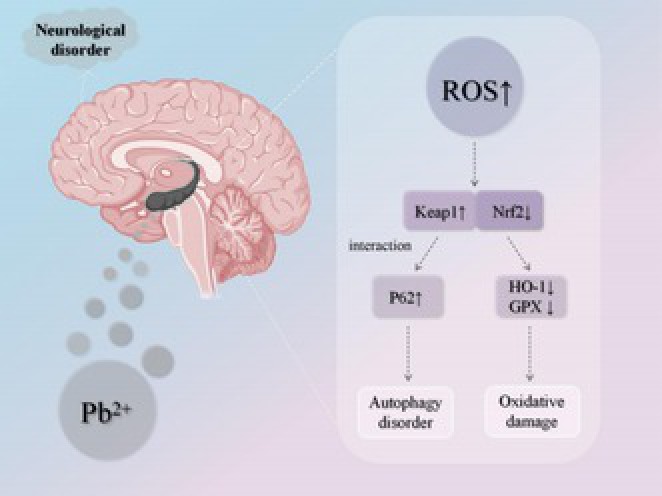

## 1 Introduction

Lead (Pb) is a widely existing environmental toxic pollutant. Environmental exposure to Pb primarily occurs through the ingestion or inhalation of contaminated dust, water, and food sources. The sources of contamination include glazed catering appliances, painted toys, cosmetics, Pb mining and smelting activities, as well as electronic waste disposal [1]. It is important to note that there is no safe threshold for Pb in the human body, and prolonged Pb exposure can result in irreversible damage. A recent report by UNICEF's Institute for Health Indicators and Evaluation (IHME) highlights that long‐term health consequences associated with Pb exposure contribute to over 902,000 deaths worldwide [2]. Consequently, the potential health hazards stemming from Pb exposure continue to be a significant concern within public health.

Pb exposure is considered a significant risk factor for the development of neurodegenerative diseases. By competitively inhibiting the absorption of essential minerals like calcium, Pb can penetrate the blood–brain barrier and accumulate in the brain, leading to neurotoxic effects [3]. Autopsy findings have revealed higher levels of Pb in both the brain and cerebrospinal fluid of individuals with neurodegenerative diseases compared to healthy individuals [4]. A cohort study conducted by Duke University in the United States demonstrated that individuals exposed to leaded gasoline during childhood exhibited diminished cognitive function in middle age and structural alterations such as reduced hippocampal volume [5]. Our previous research indicated that school‐age children residing in the Pb‐zinc mining areas of Guangxi, despite not being diagnosed with Pb poisoning, experienced impaired thyroid secretion function and decreased IQ levels due to low‐dose Pb exposure [6]. These studies strongly support the notion that environmental Pb exposure contributes to the onset of neurodegenerative diseases. However, the precise mechanisms by which Pb exposure induces neurological dysfunction and leads to cognitive impairment remain unclear.

As is well known, Pb exposure could disrupt the dynamic balance between oxidative active substances and the antioxidant defense system [7]. The Nuclear factor erythroid 2‐related factor 2 (Nrf2)/Kelch‐like ECH‐associated protein 1 (Keap1) is a classical signaling pathway that plays a central role in antioxidative stress and cell protection. This pathway is a focal point in the study of neurodegenerative diseases [8]. Nrf2 is a key regulator of endogenous antioxidative stress, containing seven (Neh1‐7) Nrf2‐ECH homologous functional domains. Among these, the Neh2 domain can bind to Keap1 through DLG and ETGE motifs [9]. Under normal physiological conditions, Keap1‐Cullin3‐E3 ubiquitin ligase targets the N‐terminal Neh2 domain of Nrf2 and promotes ubiquitination. The ubiquitinated Nrf2 is then delivered to the proteasome for degradation. When exposed to oxidative stress conditions such as mild reactive oxygen species (ROS) levels, the conformational change of Keap1‐Cul3‐E3 ubiquitin ligase interferes with Nrf2 ubiquitination. Subsequently, Nrf2 is translocated to the nucleus and binds to antioxidant response elements ARE to induce the expression of a series of cellular antioxidant protective genes, such as heme Oxygenase‐1 (HO‐1), glutathione peroxidase (GPx), etc. [10]. A recent study in a mouse model of Alzheimer's disease demonstrated significantly reduced expression of the Nrf2 gene, decreased levels of the downstream antioxidant proteins (HO‐1 and GSH), and increased expression of the Keap1 gene [11]. In vivo experiments have shown that in animal models of Parkinson's disease, by activating Nrf2 and interfering with the interaction between Nrf2 and Keap1, the related oxidative damage can be restored [12]. Long‐term Pb exposure can induce oxidative stress in rat brain [13, 14]. Meanwhile, numerous studies have shown that Pb exposure leads to a decrease in the mRNA and protein expression of Nrf2 in the brain tissues of rodents [15, 16]. However, further explanation is required regarding the alterations in Nrf2/Keap1 related to elevated levels of ROS caused by long‐term Pb exposure.

Autophagy is a self‐eating phenomenon widely present in eukaryotic cells that degrades misfolded proteins or damaged organelles through fusion with lysosomes. Numerous studies have provided evidence for the crucial role of the autophagolysosomal pathway in mediating oxidative stress [14, 17]. Prolonged and severe oxidative stress can lead to mitochondrial dysfunction, triggering rapid recruitment of ubiquitin E3 enzyme parkin by Pink1. This interaction is recognized by the autophagy protein P62, which subsequently localizes to autophagosome lysosomes via LC3 for degradation [18]. However, impaired autophagy function can result in P62 accumulation and loss of binding protein functionality [19]. P62 competes with Nrf2 for Keap1 binding, thereby influencing both autophagy occurrence and Nrf2 regulation by Keap1 [20, 21]. Studies have shown that long‐term Pb exposure promotes disruptions in brain autophagy. Pb exposure can enhance P62 accumulation in rats brains while impairing the modification and processing of LC3I into LC3II through the ubiquitin‐like system involving Atg7 and Atg3. These disturbances ultimately lead to compromised learning and memory abilities [22].

Therefore, we conducted investigations to examine the impact of long‐term Pb exposure on neuronal antioxidant capacity and autophagy through both in vivo and in vitro experiments. Our objective was to further elucidate the underlying mechanism of neurological dysfunction induced by Pb exposure and identify potential therapeutic targets for cognitive impairment induced by Pb. We discovered that Pb exposure disrupts the P62/Nrf2/Keap1 signaling pathway, thereby mediating neurodegeneration. However, treatment with the antioxidant NAC could ameliorate these alterations. This suggests that, besides chelating Pb, enhancing resistance to oxidative damage is also necessary for the clinical management of Pb neurotoxicity.

## 2 Materials and Methods

### 2.1 Animals and Experimental Design

Four‐week‐old male Sprague–Dawley (SD) rats, weighing 98.31 ± 8.58 g, were obtained from the Experimental Animal Center of Guangxi Medical University [SCXKG2020‐0003]. They were housed in a specific pathogen‐free (SPF) environment with a 12‐h light–dark cycle and ad libitum access to food and water. The experimental procedures adhered to the guidelines established by the Animal Protection and Experiment Committee of Guangxi Medical University and received approval from the Animal Protection and Use Committee of Guangxi Medical University.

Rats were randomly divided into two groups (Control and Pb‐exposed groups), each consisting of 10 individuals. Rats in the Pb group received intraperitoneal injections (i.p.) of 2 mg/kg Pb(C_2_H_3_O_2_)_2_ once daily for 5 days/week over 12 weeks to induce sub‐chronic Pb exposure. The control group received an equal volume of saline via i.p. After 12 weeks of Pb exposure, the Morris water maze test was conducted to evaluate learning and spatial memory performance in rats. The dose of Pb(C_2_H_3_O_2_)_2_ was chosen based on our previous study [23].

### 2.2 Cell Culture

SH‐SY5Y cell line was obtained from the China Center for Type Culture Collection (CCTCC, Wuhan University, China), and cultured in a medium containing 10% FBS, 100 U/mL penicillin, 0.1 mg/mL streptomycin, MEM, and DMEM/F12 (1:1). Cells were maintained under humidified conditions at 37°C with 5% CO_2_.

### 2.3 Morris Water Maze (MWM) Test

The MWM test was conducted to assess the learning and memory abilities of rats [24]. Rats were released into the maze from four different directions to find a hidden platform. Swimming distance and escape latency were recorded. Rats failing to locate the platform within 90 s during training were guided toward it. After 5 days of training, the platform was removed, and spatial memory ability was evaluated by recording how many times rats crossed the probe within 120 s.

### 2.4 Histopathology Examination of Hippocampus

Hematoxylin and eosin (HE) staining, as well as Nissl staining, were conducted in accordance with previously established protocols [25]. Samples were fixed, dehydrated, embedded in wax blocks, and sectioned into 5 μm‐thin slices. HE or Nissl staining was performed to observe morphological alterations.

### 2.5 Determination of pb Concentration

The MARS6 Classic Microwave Digestion System was used to digest 50 mg of hippocampus and 300 μL of whole blood with 5 mL of nitric acid. Subsequently, the samples were evaporated on a graphite heating plate at 150°C until the liquid volume was reduced to approximately 0.5 mL. Samples were then cooled to room temperature and diluted with deionized water to reach a final volume of 10 mL. Pb levels were analyzed using Inductively Coupled Plasma Mass Spectrometry (ICP‐MS, Nexion 300D, USA).

### 2.6 Cytotoxicity Test

The cytotoxicity test was performed following previously described protocols [26]. SH‐SY5Y cells were seeded at a density of 8000 cells per well in a 96‐well plate and incubated for 24 h under controlled humidified conditions with 5% CO_2_ at 37°C. Subsequently, cells were exposed to media containing Pb concentrations of 0, 1, 5, 25, 50, 100, and 200 μmol/L for an additional 24 h. Each well was treated with a solution of Cell Counting Kit‐8 (CCK‐8, C0005, Targetmol, China), followed by incubation for two more hours. Absorbance at 450 nm was measured using an enzyme‐linked immunosorbent assay reader.

### 2.7 F‐Actin Staining

F‐actin staining was conducted following previously described protocols [27]. Briefly, the prepared cells were fixed with a 4% formaldehyde solution at room temperature for 10 min. They were then washed three times with PBS containing 0.1% Triton X‐100, each time for 5 min. Actin‐Tracker Red (1:100, C2205S, Beyotime, China) was diluted in PBS containing 5% BSA and 0.1% Triton X‐100, followed by incubation at room temperature in the dark for 40 min. Afterward, the cells were washed twice with PBS for 5 min and directly observed under a fluorescence microscope.

### 2.8 Measurement of ROS

The measurement of the ROS procedure was conducted according to the previous study [24]. When Pb was exposed to Pb for 23.5 h, the positive control wells were treated with 4‐butyl hydrogen peroxide (Rosup) for 30 min. After that, SH‐SY5Y cells were treated with 10 μmol/L 2,7‐dichlorodihydrofluorescein diacetate (DCFH‐DA, S0033s, Beyotime, China) for 1 h at 37°C in the dark. The detection was performed strictly following the manufacturer's instructions and previous studies. Fluorescence visualization was conducted using the EVOS fluorescence microscopy imaging system (Thermo, USA).

### 2.9 Detection of GPx Activity

The detection of GPx activity procedure was conducted according to the previous study [28]. Cells were scraped and collected, followed by one wash with PBS. Cell lysis buffer (P0013, Beyotime, China) was used to lyse the cells, followed by centrifugation at 4°C and 12,000 *g* for 10 min. The supernatant was used for enzyme activity determination. Protein concentration was measured using the BCA assay kit (P0012, Beyotime, China). In a 96‐well plate, 50 μL of the test sample was added (with an additional 50 μL of Cell lysis buffer in the blank control well), followed by the addition of 40 μL of GPx detection working solution (S0056, Beyotime, China). Subsequently, each well received 10 μL of a 30 mM hydrogen peroxide reagent solution and was thoroughly mixed. Absorbance at 340 nm was measured using an enzyme‐linked immunosorbent assay reader. Results were standardized against protein concentration.

### 2.10 Immunofluorescence Assays

Cells were fixed with 4% paraformaldehyde at room temperature for 10 min. Subsequently, permeabilization was performed by incubating the cells with PBS containing 0.1% Triton X‐100 at room temperature for 10 min. Following this, the cells were blocked with 5% BSA at room temperature for 30 min. Primary antibodies, including anti‐Nrf2 (1:500, Cell Signaling Technolog (CST), #12721) and anti‐LC3 (1:100, CST, #12741) were incubated overnight with the samples at 4°C. After three washes with PBS for 5 min each time, appropriate dilutions of anti‐rabbit IgG (1:1000, Alexa Fluor 488 Conjugate, CST, #4412S) or anti‐rabbit IgG (1:1000, Alexa Fluor 594 Conjugate, CST, #8889) in PBS were added to the samples and incubated for 1 h in a dark environment at room temperature. The samples were then stained with DAPI for 5 min after being rinsed three times in PBS for 5 min each time. Finally, the samples were observed under a Ti2‐U inverted fluorescence microscope (Nikon Eclipse, Japan).

The procedures of animal brain tissue immunofluorescence were conducted according to the previous study [29]. Brain specimens were collected, fixed, dehydrated, embedded in wax blocks, and sliced into 5 μm‐thick sections. After deparaffinization and antigen retrieval procedures, the slices underwent a blocking process with 5% BSA for half an hour before being subjected to overnight incubation at 4°C using Rabbit anti‐Nrf2 (1:500, CST, #12721). After three rinses in PBS for 5 min each time, the sections were exposed to anti‐rabbit IgG (HRP) at room temperature for 1 h, followed by FITC‐Tyramide (TSA) (Servicebio, G1222) treatment in darkness for 10 min. After the tyramide reaction, slides were washed and treated with 3% H_2_O_2_ for 15 min to quench HRP activity. To further ensure specificity, a mild stripping buffer (pH 2.0 glycine‐HCl) was used between sequential rounds to remove previous antibodies. Subsequently, the sections underwent antigen retrieval once again and were then incubated with Rabbit anti‐Keap1 (1:200, CST, #8047) or Rabbit anti‐P62 (1:100, CST, #39786). After three rounds of PBS rinses, the sections were subjected to incubation with anti‐rabbit IgG (HRP) at room temperature for 1 h. CY3‐TSA (Servicebio, G1223) or iF647‐TSA (Servicebio, G1232) was then applied in darkness for 10 min. After three rounds of PBS rinses, the sections were incubated with DAPI for 5 min. The sections were examined using the microscope slide scanner (Pannoramic DESK, Hungary). The procedures were conducted according to the previous study.

### 2.11 Western Bloting

The western blot procedures were conducted according to the previous study [29]. The antibodies used in this study included anti‐β‐actin (1:1000, CST, #4967), anti‐GAPDH (1:1000, CST, #2118), anti‐Nrf2 (1:1000, CST, #12721), anti‐Keap1 (1:1000, CST, #8047), anti‐HO‐1 (1:1000, CST, #43966), anti‐LC3 (1:1000, CST, #12741), anti‐P62 (1:1000, CST, #39786), and anti‐Tau (1:1000, CST, #4019). Additionally, the secondary antibodies of anti‐rabbit IgG (1:3000, CST, #7074) and anti‐mouse IgG (1:3000, CST, #7076) were used. The protein bands' optical density was measured using an iBright Western Blot imaging system (iBright Fl1000, Thermo).

### 2.12 Statistical Analysis

The data analysis was performed using GraphPad Prism, version 9.5. All experiments were repeated three or four times independently. All data were subject to tests for normality. Satisfy the normal distribution, and use student's t‐test or one‐way ANOVA analysis. We employed Dunnett's multiple comparisons test for pairwise comparisons. Repeated measures analysis of variance was used for MWM Test data. Non‐normal data used corresponding non‐parametric tests. Results are presented as means with standard deviations, and statistical significance was determined at *p* < 0.05.

## 3 Results

### 3.1 Pb Exposure Causes Neuronal Dysfunction and Neurobehavioral Disorders

**FIGURE 1:**
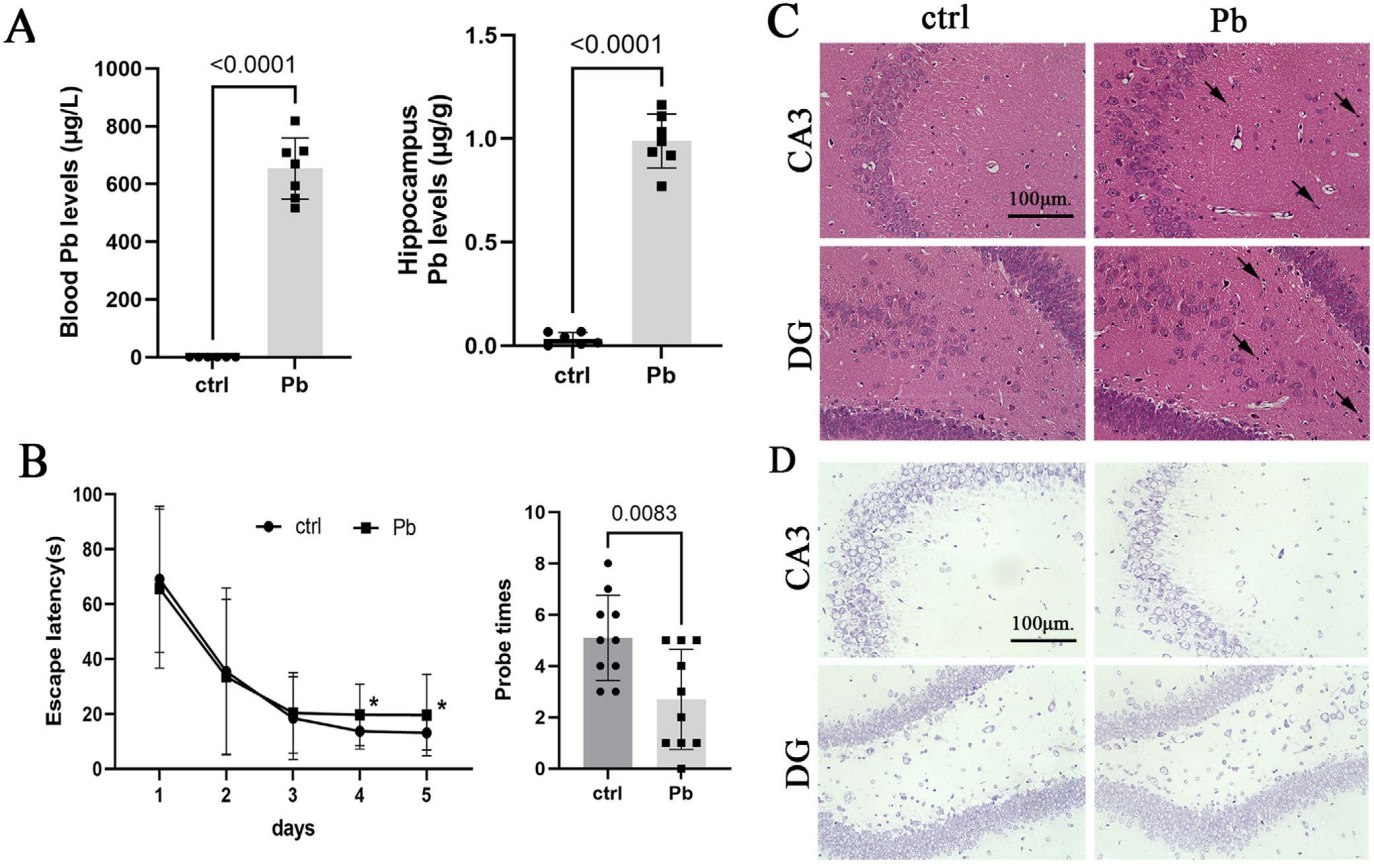
Pb exposure causes neurofunctional impairments in rats. (A) Pb levels in the Blood and hippocampal of rats. (B) Morris water maze test was used to assess the learning and memory abilities of rats; Left: Escape latency; Right: Number of probe times within 2 min. (C) HE staining of the hippocampus in lead‐exposed rats. (D) Nissl staining of the hippocampus in Pb‐exposed rats. Repeated measures analysis of variance was used for MWM Test data. Other quantitative data was presented as mean ± SD (*n* ≥ 7) and analyzed by Student's *t*‐test.

After Pb exposure for 12 weeks, both blood and hippocampal Pb levels in rats were significantly increased (Figure 1A), indicating the successful establishment of the Pb exposure model. Compared to the control group, rats exposed to Pb showed a significant increase in escape latency on the fourth and fifth days, as well as a decrease in platform crossing times (Figure 1B). HE staining revealed that the hippocampal neurons in the control group were arranged regularly, with dense cell nuclei and no obvious cell loss. In contrast, neurons in the CA3 and DG regions of Pb‐exposed rats exhibited a loose tissue pattern, increased nuclear condensation, and less distinct cell nuclei (Figure 1C). The nissl bodies exhibit a blue hue in Nissl staining; however, under pathological conditions, their quantity diminishes, resulting in a lighter or even complete absence of the blue color. Our results showed that nissl staining indicated lighter staining and fewer nissl bodies in neurons stained with Pb compared to the control group, suggesting impaired neural function due to long‐term Pb exposure (Figure 1D).

### 3.2 Disruption of the Keap1/Nrf2 Signaling Pathway Mediates Oxidative Damage in pb‐Exposed Neurons

**FIGURE 2:**
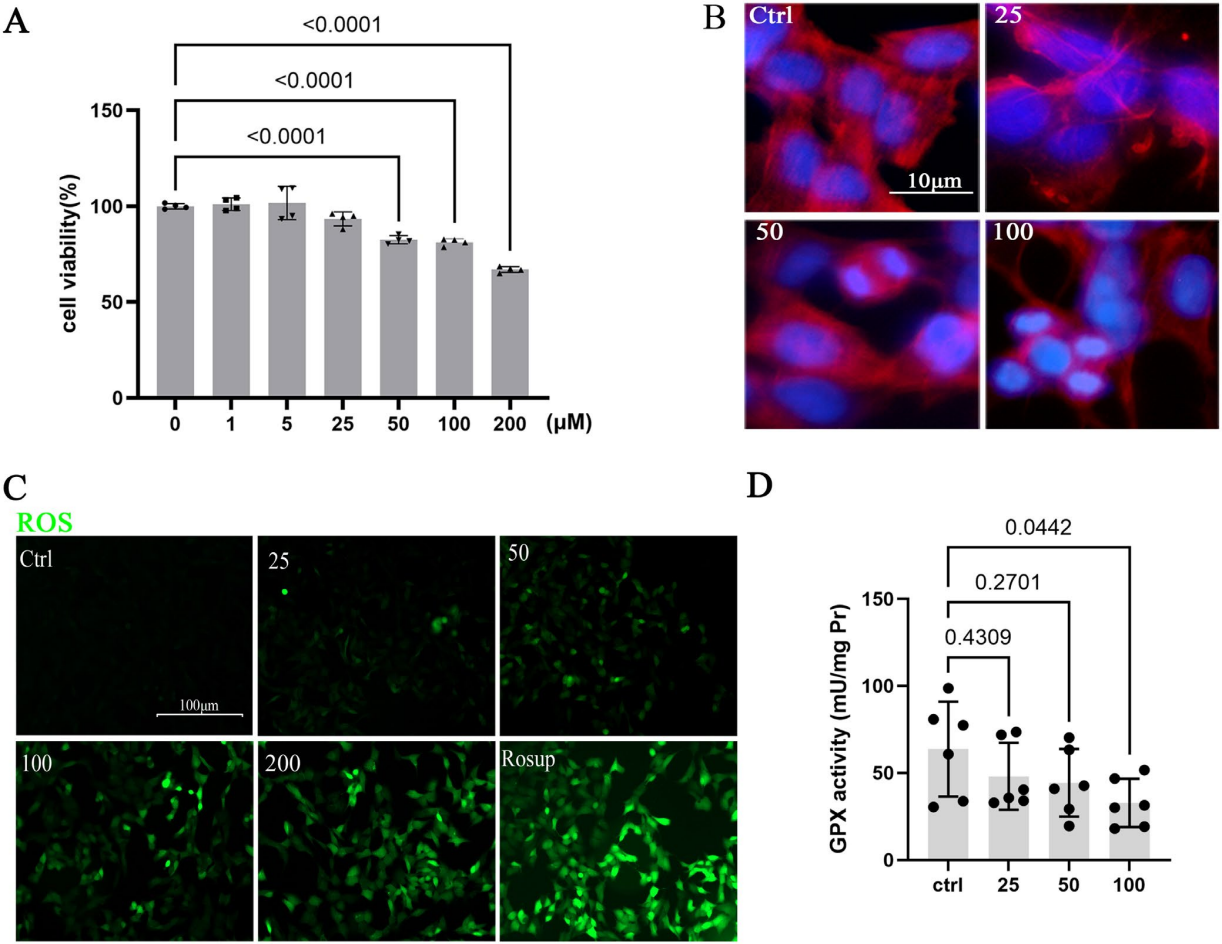
Pb exposure induces oxidative damage in SH‐SY5Y cells. SH‐SY5Y cells were exposed to Pb at concentrations of 0, 25, 50, and 100 μmol/L. (A) Cell viability of Pb‐exposed SH‐SY5Y cells. (B) Phenotypic changes in Pb‐exposed SH‐SY5Y cells at different concentrations; Red: F‐actin; Blue: DAPI. (C) Detection of ROS levels using DCFH‐DA probe in Pb‐exposed SH‐SY5Y cells; Green: ROS. (D) Measurement of GPx activity in Pb‐exposed SH‐SY5Y cells. Quantitative data was presented as mean ± SD (*n* > 3) and analyzed by one‐way ANOVA.

**FIGURE 3:**
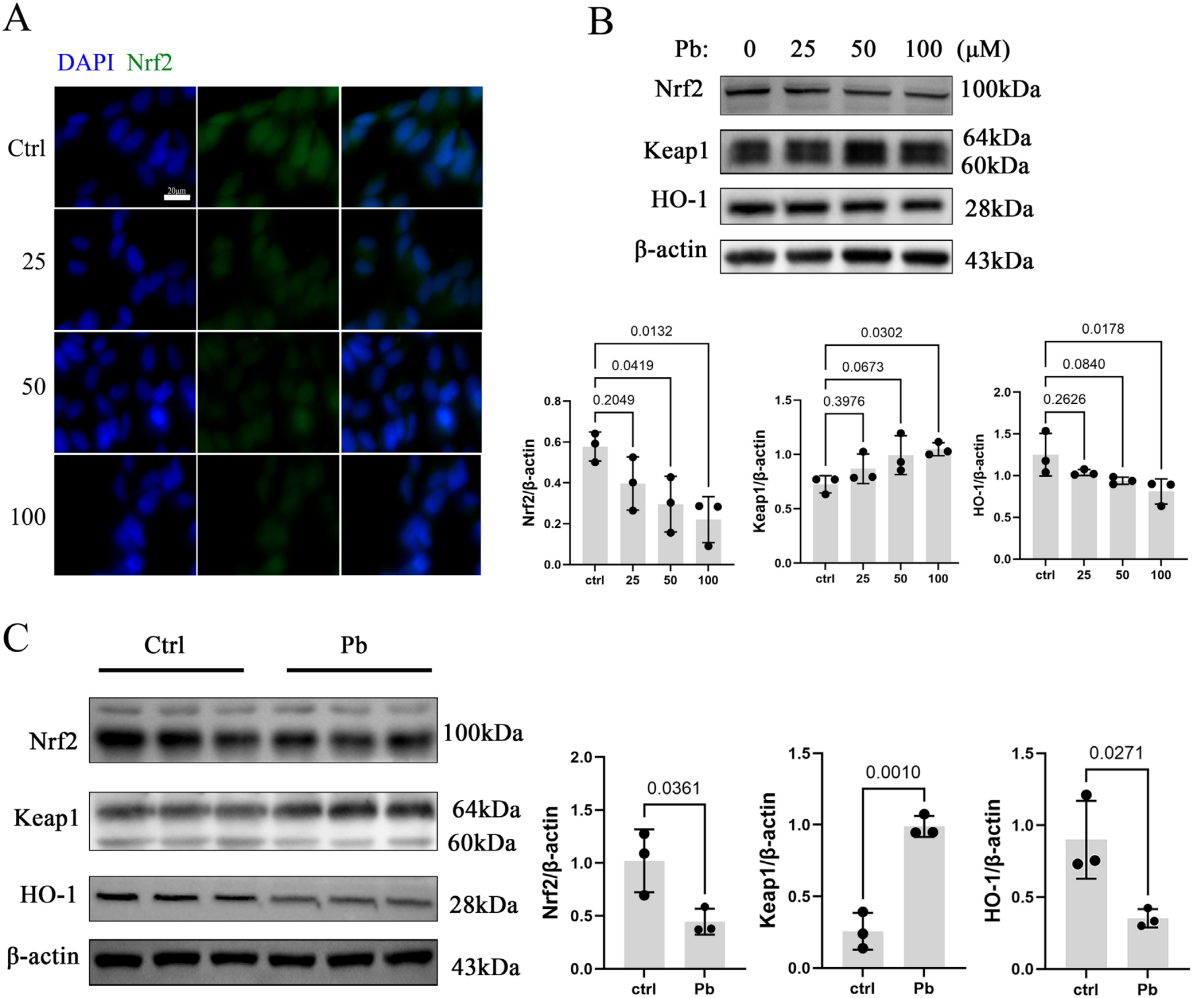
Disruption of the Keap1/Nrf2 signaling pathway mediates Pb‐induced cellular oxidative damage. (A) Immunofluorescence detection of Nrf2 expression in Pb‐exposed SH‐SYS5Y cells; Green: Nrf2; Blue: DAPI. (B) Detection of Nrf2, Keap1, and HO‐1 proteins by Western blot in Pb‐exposed SH‐SYS5Y cells. (C) Detection of Nrf2, Keap1, and HO‐1 protein expression in the hippocampus of Pb‐exposed rats. Quantitative data was presented as mean ± SD (*n* = 3) and analyzed by one‐way ANOVA or Student's *t*‐test.

To elucidate the relationship between Pb‐induced neurotoxicity and oxidative stress, we employed SH‐SY5Y cells and a rat model of Pb exposure. Treatment of SH‐SY5Y cells with varying concentrations of Pb for 24 h resulted in a significant decrease in cell viability at 50 μM, with a more pronounced decrease as the concentration increased (Figure 2A). Subsequent toxicological experiments were conducted at concentrations of 25 μM (no significant changes in cell viability), 50 μM (initial damage observed), and 100 μM (obvious damage occurred). F‐actin staining results showed that there were noticeable phenotypic changes in the cells, such as cell shrinkage and nuclear condensation at 50 μM, which became more apparent at 100 μM (Figure 2B). DCFH‐DA probe detection revealed that ROS levels were increased after Pb exposure in a dose‐dependent manner (Figure 2C). Furthermore, the activity of the GPx antioxidant enzyme decreased with increasing concentration of Pb (Figure 2D). Additionally, immunofluorescence results demonstrated reduced levels of nuclear and cytoplasmic Nrf2 protein expression (Figure 3A). Western blot analysis revealed decreased expression of Nrf2 and its downstream target HO‐1, accompanied by increased Keap1 expression (Figure 3B). Consistent findings were also observed in hippocampal protein expression levels in Pb‐exposed rats (Figure 3C), indicating that neuronal oxidative damage is induced by Pb exposure while disrupting the Nrf2/Keap1 pathway.

### 3.3 Pb Exposure Causes Neurocellular Autophagy Disorders and Mediate Neurodegeneration

**FIGURE 4:**
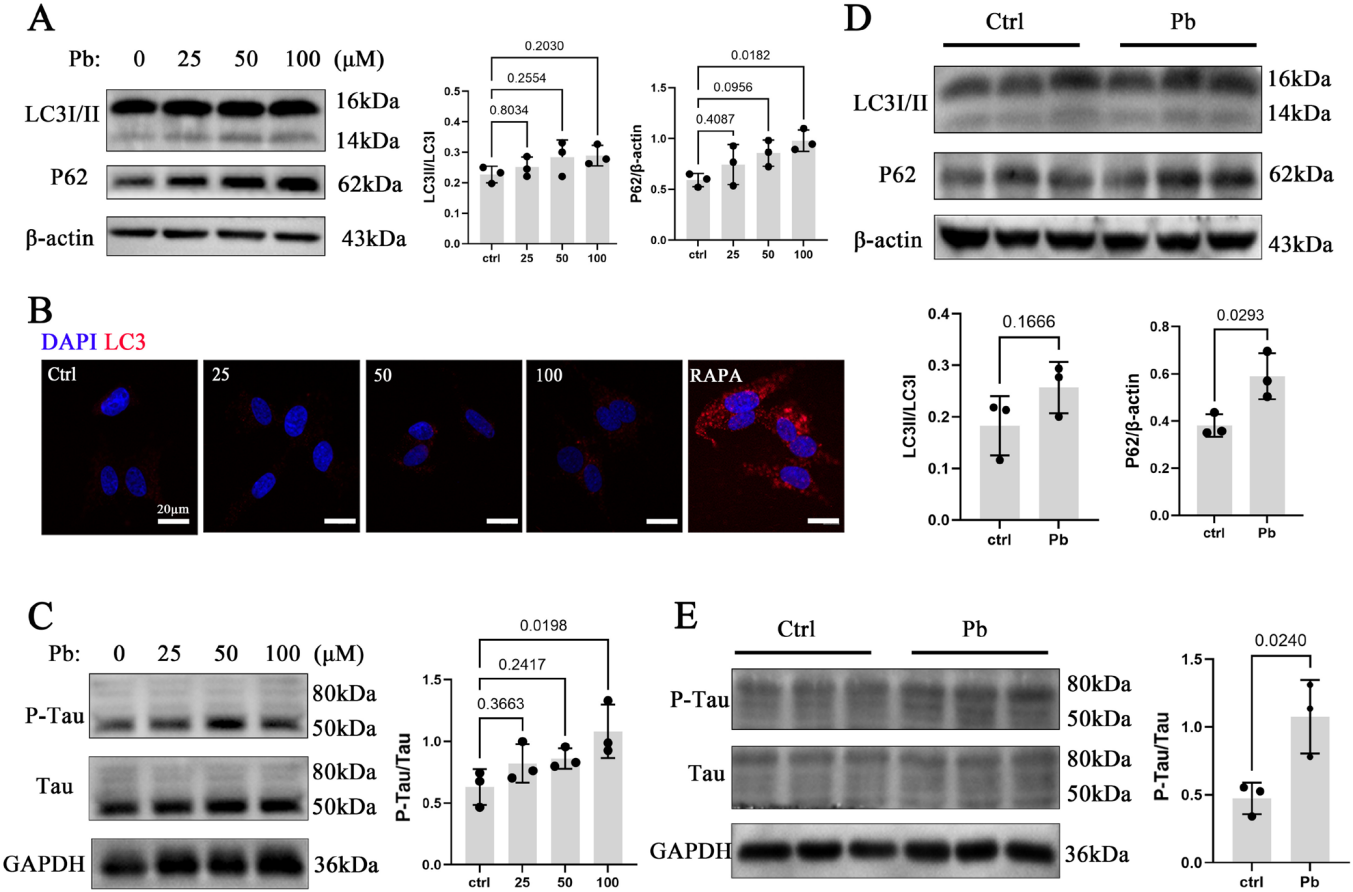
Pb exposure can cause neurocellular autophagy disorders and mediate neurodegeneration. (A) Western bloting analysis of the expression levels of LC3 and P62 proteins in Pb‐exposed SH‐SY5Y cells. (B) Immunofluorescence detection of LC3 protein levels. Red: LC3; Blue: DAPI. (C) Western blot analysis of Tau protein expression levels in SH‐SY5Y cells exposed to Pb. (D) Western blot analysis of the expression levels of LC3 and P62 proteins in the hippocampus of Pb‐exposed rats. (E) Western blot analysis of Tau protein expression in the hippocampus of Pb‐exposed rats. Quantitative data was presented as mean ± SD (*n* = 3) and analyzed by one‐way ANOVA or Student's *t*‐test.

To further investigate neuronal autophagy following oxidative damage caused by Pb exposure, both in vitro and in vivo studies were conducted. The results showed no significant increase in the ratio of LC3II/LC3I, a marker protein for autophagy, in SH‐SY5Y cells after Pb exposure (Figure 4A). However, the protein expression of P62 increased with escalating concentrations of Pb (Figure 4A). Similarly, immunofluorescence analysis demonstrated no notable elevation in the expression of LC3 protein (Figure 4B), consistent with in vivo findings (Figure 4D). Importantly, there was a substantial upregulation observed in the protein expression of Tau, an AD‐related protein, following Pb exposure (Figure 4C,E), suggesting that Pb exposure disrupts autophagy and mediates neurodegeneration.

### 3.4 P62 Interacts With the Keap1/Nrf2 Axis in the Hippocampus of pb‐Exposed Rats

**FIGURE 5:**
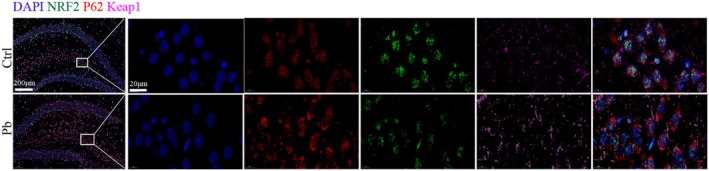
P62 crosstalks with the Nrf2/Keap1 axis in the hippocampus of Pb‐exposed rats. Immunofluorescence detection of Nrf2, P62, and Keap1 expression levels and co‐localization in the hippocampus of Pb‐exposed rats. Green: Nrf2; Red: P62; Blue: DAPI; Purple: Keap1.

To further elucidate the relationship between Nrf2/Keap1 and the autophagy protein P62, immunofluorescence detection was performed on the hippocampus of Pb‐exposed rats. The results indicated that there was co‐localization of Nrf2, Keap1, and P62 proteins in the Pb‐exposed group. The Nrf2 protein expression decreased, and the P62 and Keap1 protein expressions increased in the Pb‐exposed group (Figure 5), consistent with the results of Western blot. This suggests that the Keap1 protein binds to P62, inhibiting its localization to autolysosomes and thus suppressing autophagy.

### 3.5 Inhibition of Oxidative Stress Alleviates pb‐Induced Neurological Dysfunction via Improving the P62/Nrf2/Keap1 Pathway

**FIGURE 6:**
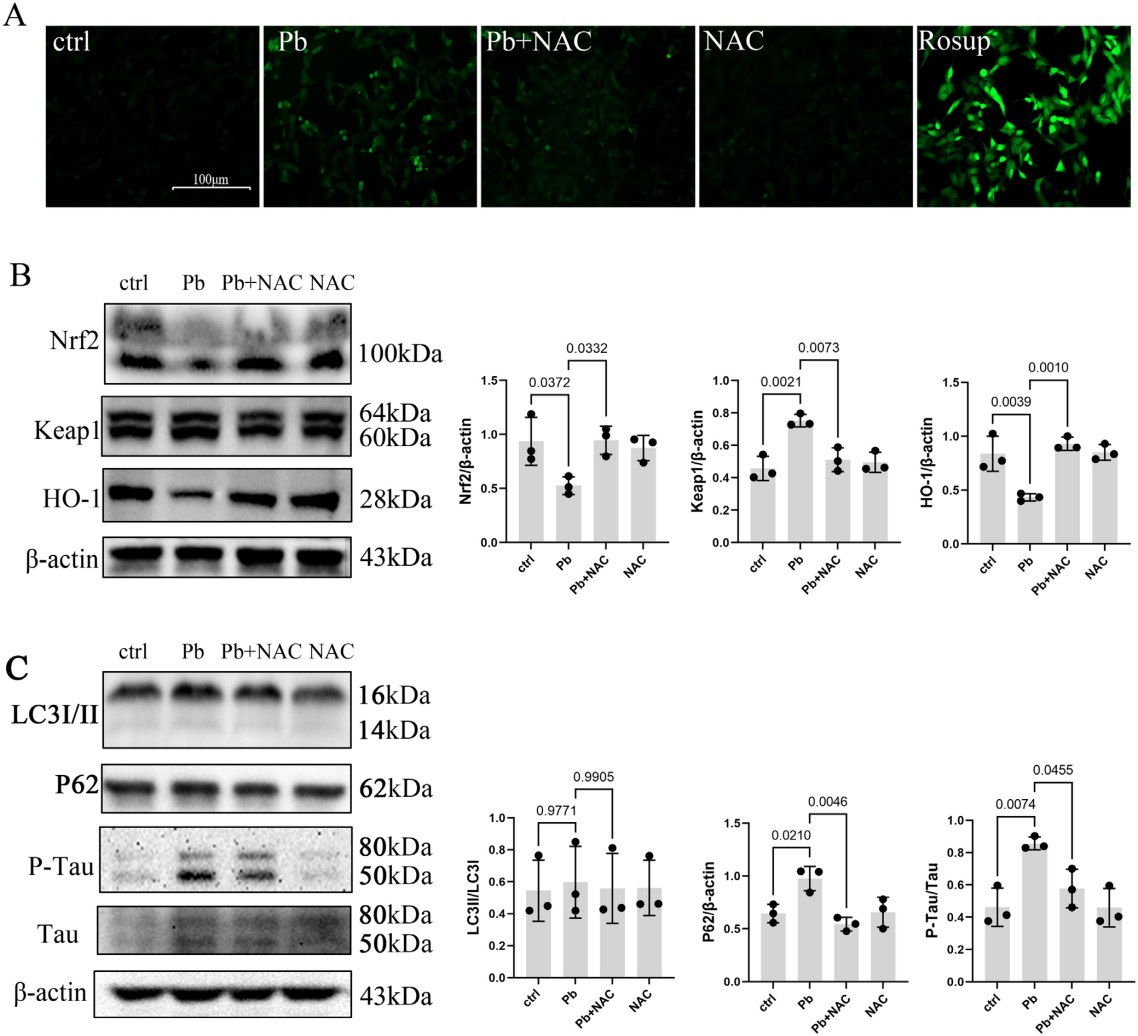
Inhibition oxidative stress alleviates Pb‐induced neurodegeneration via improving the P62/Nrf2/Keap1 pathway. SH‐SY5Y cells were pre‐treated with NAC for 1 h followed by exposure to 50 μM Pb for 24 h. (A) DCFH‐DA probe was used to detect ROS levels; Green: ROS. (B) Western blot analysis was performed to determine Nrf2, Keap1, and HO‐1 protein expressions. (C) Western blot analysis was conducted to assess LC3, P62, and Tau protein expressions in the hippocampus of Pb‐exposed rats. Quantitative data was presented as mean ± SD (*n* = 3) and analyzed by one‐way ANOVA.

To further investigate the role of oxidative reactions, disruption of the Nrf2/Keap1 pathway, and inhibition of autophagy caused by Pb exposure, SH‐SY5Y cells were treated with the antioxidant N‐acetyl cysteine (NAC). The results demonstrated that treatment with NAC effectively reduced ROS levels in Pb‐exposed SH‐SY5Y cells (Figure 6A). Moreover, NAC ameliorated the downregulation of protein expressions of Nrf2 and HO‐1 induced by Pb while concurrently decreasing Keap1 expression (Figure 6B). Furthermore, NAC decreased P62 protein expression without significantly altering the LC3II/LC3I ratio (Figure 6C), suggesting that autophagy may not be necessary in cells after antioxidant treatment, possibly due to the absence of oxidative damage.

### 3.6 Activation of Nrf2 and Promotion of Autophagy Alleviate pb‐Induced Neurological Dysfunction

**FIGURE 7:**
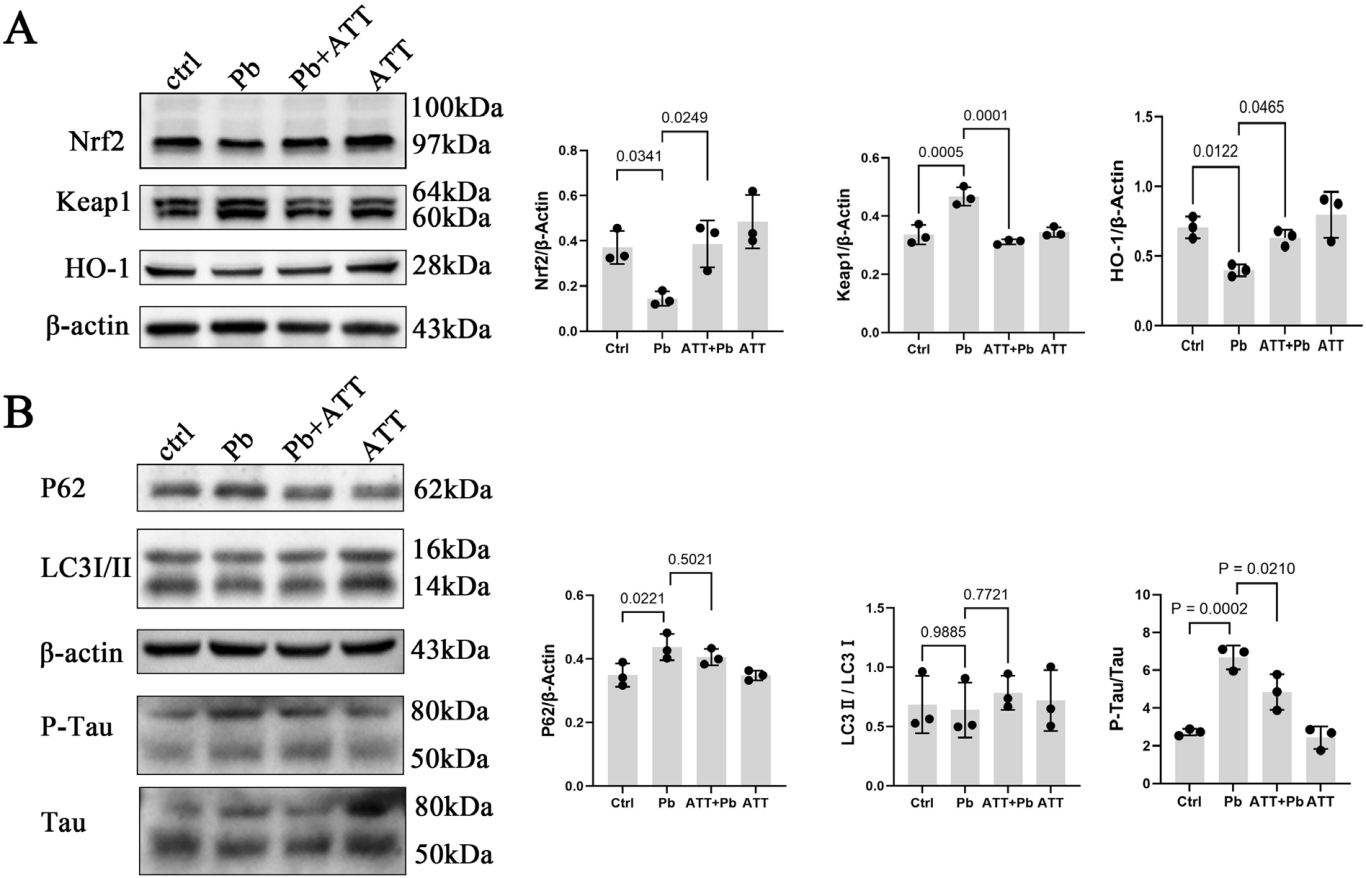
Nrf2 activator Artemisitene alleviate Pb‐induced neurological dysfunction. SH‐SY5Y cells were pre‐treated with 2 μM Artemisitene (ATT) for 1 h followed by exposure to 50 μM Pb for 24 h. (A) Detection of Nrf2, Keap1, and HO‐1 proteins by Western blot. (B) Detection of P62, LC3, P62, P‐Tau and total Tau proteins by Western blot. Quantitative data was presented as mean ± SD (*n* = 3) and analyzed by one‐way ANOVA.

To activate Nrf2, SH‐SY5Y cells exposed to Pb were pretreated with the Nrf2 activator Artemisitene (ATT) (MedChemExpress, MCE, HY‐122550) [30]. The results demonstrated that ATT pretreatment significantly increased Nrf2 expression and heme oxygenase‐1 (HO‐1) levels, while decreasing Keap1 expression compared to the Pb‐exposed group (Figure 7A). Additionally, phosphorylated Tau (P‐Tau) expression was notably reduced. These findings suggest that enhancing Nrf2 activity potentiates the antioxidant capacity of SH‐SY5Y cells, partially alleviating lead‐induced neurodegenerative pathology. Furthermore, ATT pretreatment led to a slight reduction in P62 expression and a modest increase in the LC3II/LC3I ratio compared to the lead‐exposed group, although the difference did not reach statistical significance (Figure 7B). This observation implies that Nrf2 activation may mitigate lead‐triggered autophagy impairment.

**FIGURE 8:**
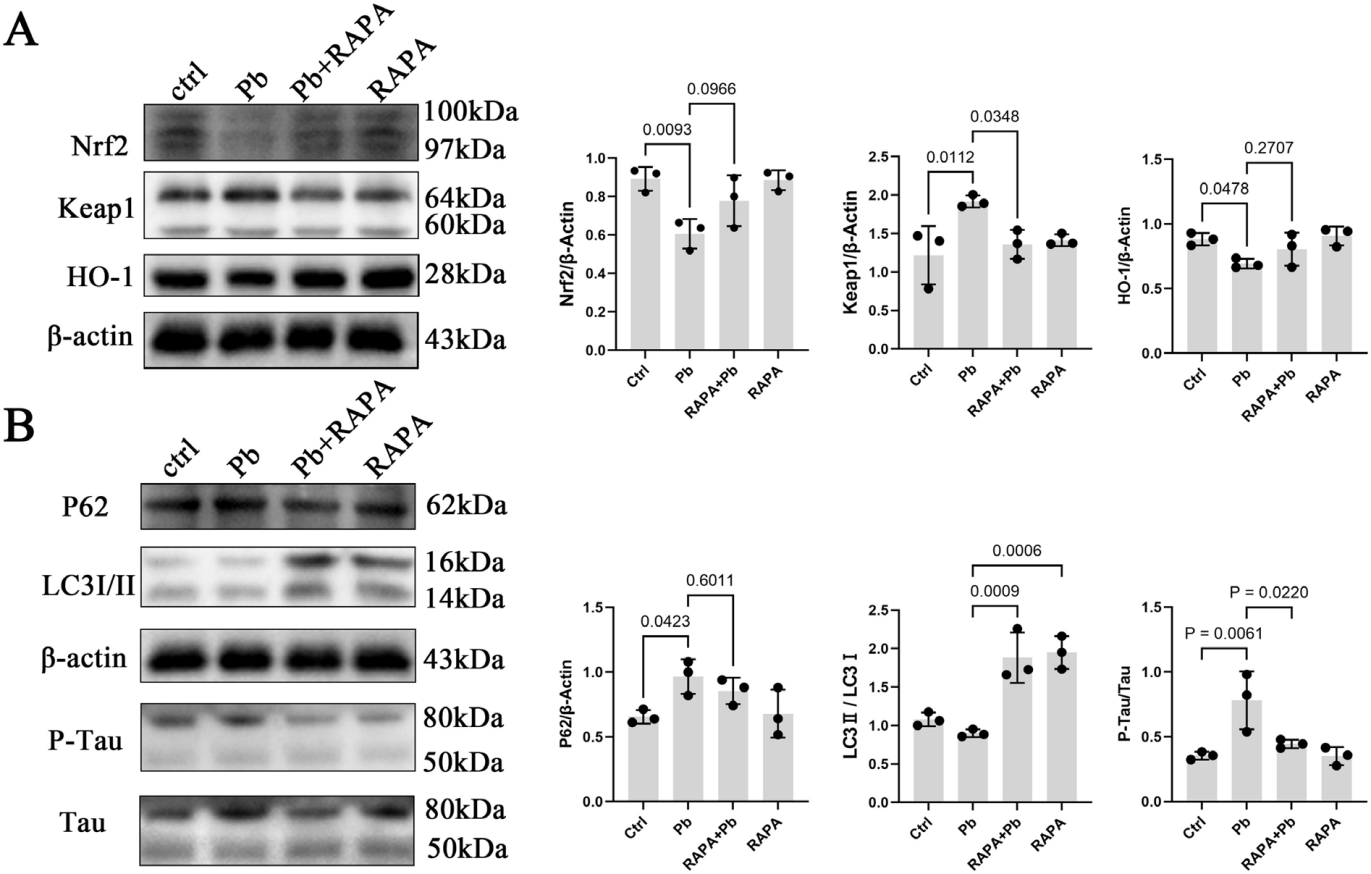
Rapamycin alleviates lead‐induced neurological dysfunction by promoting autophagy. SH‐SY5Y cells were pre‐treated with 1 μM Rapamycin (RAPA) for 4 h followed by exposure to 50 μM Pb for 24 h. (A) Detection of Nrf2, Keap1, and HO‐1 proteins by Western blot. (B) Detection of P62, LC3, P‐Tau and total Tau proteins by Western blot. Quantitative data was presented as mean ± SD (*n* = 3) and analyzed by one‐way ANOVA.

To enhance cellular autophagy, SH‐SY5Y cells exposed to Pb were pretreated with the autophagy activator Rapamycin (RAPA) (MCE, HY‐10219) [31]. Compared to the Pb‐exposed group, RAPA pretreatment resulted in a reduction in P62 expression (albeit statistically insignificant) and an elevated LC3II/LC3I ratio, suggesting that RAPA promotes autophagic flux in lead‐exposed SH‐SY5Y cells (Figure 8B). Furthermore, RAPA pretreatment downregulated Keap1 expression and modestly increased Nrf2 and heme oxygenase‐1 (HO‐1) levels in lead‐exposed cells, though these differences were not pronounced (Figure 8A). Concurrently, phosphorylated Tau (P‐Tau) expression was reduced (Figure 8B), indicating that autophagy induction alleviates lead‐induced neurotoxicity.

Notably, both Nrf2 activation by ATT and RAPA‐mediated P62 reduction were associated with decreased Keap1 protein levels. These findings collectively highlight the critical role of the P62/Nrf2/Keap1 signaling pathway in mitigating lead‐induced neurodegenerative dysfunction.

## 4 Discussion

Pb exposure induces cognitive impairments and contributes to neurodegenerative diseases by disrupting cellular redox homeostasis and protein clearance pathways. Pb exposure induces cognitive impairments and contributes to the development of neurodegenerative diseases. The present study suggests that Pb exposure triggers a significant production of ROS, upregulates Keap1 expression, degrades Nrf2, hinders normal nuclear translocation of Nrf2, and inhibits the expression of downstream antioxidant protein HO‐1 and GPx, resulting in oxidative damage to neurons. Due to the decrease in Nrf2, Keap1 expression increases, which may lead to enhanced binding of P62 to Keap1, thereby impeding P62 from fulfilling its autophagy function through LC3. While prior studies demonstrate that P62 competitively binds Keap1 to activate Nrf2 under physiological or acute stress conditions [32, 33], our findings reveal a distinct regulatory mechanism whereby chronic Pb exposure impairs autophagic flux, resulting in oxidative overload and damaged proteins accumulation within cells and ultimately contributing to neurodegeneration.

The brain emerges as a pivotal target organ of Pb toxicity, particularly the developing brain [34]. Multiple cohort studies have demonstrated that Pb exposure can disrupt the structural integrity of children's brains and impair cognitive abilities [35, 36]. Our preliminary research has also revealed decreased learning and memory abilities in children residing in Pb‐zinc mining areas [6]. Occupational exposure to elevated levels of Pb also increases the risk of developing amyotrophic lateral sclerosis [37]. In vivo studies have demonstrated that early‐life exposure to Pb can result in excessive deposition of Aβ amyloid and tau protein in the cerebral cortex of adult primates, mirroring pathological alterations similar to those observed in AD [38]. Moreover, childhood exposure to Pb can induce cerebral lesions in rats, characterized by an upregulation of AD‐associated proteins such as glycogen synthase kinase (GSK) and tau during advanced age [39]. A recent study suggests that Pb exacerbates the pathology of AD via mitochondrial copper accumulation, regulated by COX17 [40]. In this present study, we utilized 4‐week‐old rats that were freshly weaned and exposed to Pb for 3 months. The findings revealed that the Pb‐exposed rats exhibited a decline in their learning and memory abilities, accompanied by significant pathological changes in the brain.

The toxic effects of Pb are associated with oxidative stress [7]. Under normal physiological conditions, there exists a dynamic balance between oxidative active substances and the antioxidant defense system in the human body. Exposure to environmental Pb disrupts this balance, leading to an elevation in ROS levels, resulting in disruption and impairment of the antioxidant defense system, including GSH and GPx. Consequently, this disturbance disrupts the balance within the system, resulting in oxidative damage in various tissues and cells [41, 42]. It is worth noting that oxidative damage is also one of the key pathogenic mechanisms of neurodegenerative diseases [43]. Chronic Pb exposure can diminish the content of GPx and increase the content of malondialdehyde (MDA), a lipid peroxide product, in rodent brain tissues [34, 44]. Our research has demonstrated that Pb exposure can increase ROS levels and promote oxidative damage in neurons. The Keap1 protein acts as a sensor for ROS, is upregulated in response to ROS stimulation, and regulates the antioxidant capacity of cells through its interaction with Nrf2 [45]. Our results demonstrate that Pb exposure increases the expression of Keap1 protein in neurons. By binding to Nrf2, Keap1 facilitates its ubiquitination process [46]. Consequently, ubiquitinated Nrf2 is targeted by the proteasome for degradation. This leads to a decrease in Nrf2 protein expression and its translocation into the nucleus, culminating in reduced expression of downstream proteins such as HO‐1 and diminished GPx activity. These findings indicate that Pb exposure mediates oxidative damage to neurons through the Nrf2/Keap1 pathway.

Under conditions of oxidative stress, the intracellular autophagy‐lysosomal system can engulf and degrade damaged cellular components, thereby maintaining normal cellular function [39]. However, the autophagy pathway is often influenced by aging and external stimuli, rendering it unable to clear harmful cellular components [47, 48]. Studies have demonstrated that impaired autophagic function can contribute to the development of neurodegenerative diseases [49]. In recent years, numerous studies have revealed that Pb exposure can impair autophagic function in the nervous system, resulting in decreased learning and memory abilities in mice [50]. Pb exposure has been shown to inhibit the formation of autophagosomes in the central nervous system of zebrafish embryos, leading to neurotoxic effects [51]. Our results suggest that Pb exposure may impair the conversion of LC3‐I to LC3‐II, potentially due to disruption of the ubiquitin‐like conjugation systems involving Atg7 and Atg3, as reported in prior studies [22], impeding autophagosome formation. This inhibitory effect may serve as a negative feedback signal causing overexpression of P62 protein within cells. P62 competitively binds with Nrf2 to Keap1, preventing its recognition and regulated by autophagolysosomes [19, 20]. Nevertheless, treatment with NAC could ameliorate this alteration and reduce the expression of the AD‐related protein Tau. However, there was no increase observed in LC3‐II protein expression, possibly due to NAC inhibiting oxidative damage caused by Pb exposure on neurons, allowing for normal cell function without reliance on autophagic clearance. Pb exposure induces oxidative stress, disrupts the P62/Nrf2/Keap1 pathway, and ultimately leads to impaired neuronal function.

Enhancing autophagy and upregulating Nrf2 activity may partially alleviate Pb‐induced neurotoxicity by promoting autophagic clearance or augmenting cellular antioxidant responses. However, the protective effects of Nrf2 activation or autophagy induction appear less pronounced compared to those of NAC, which likely stems from NAC's ability to directly suppress oxidative species generation at their source. While Nrf2 activation or autophagy enhancement may improve antioxidant capacity or reduce oxidative burden, such downstream interventions might only moderately attenuate oxidative damage. Notably, our results highlight that upstream strategies targeting the suppression of oxidative species generation—such as NAC treatment—exert more robust protection against lead‐induced cellular injury. This underscores the critical importance of addressing oxidative stress at its origin to effectively mitigate Pb‐associated neurotoxicity.

In conclusion, our study demonstrates that Pb‐induced neurotoxicity primarily arises from oxidative stress, which degrades Nrf2 and upregulates Keap1, potentially promoting Keap1 binding to P62 and thereby preventing P62 from carrying out its autophagic function via LC3. Crucially, we found that interventions targeting these pathways—the antioxidant NAC, Nrf2 activator ATT, and autophagy inducer RAPA—effectively mitigate Pb's detrimental effects. This suggests that effective clinical management of Pb neurotoxicity requires not only chelating Pb but also enhancing patients' antioxidant capacity.

## Author Contributions

**Dongjie Peng:** conceptualization, methodology, software, data curation, writing – original draft, writing – review and editing. **Peiqi Wei** and **Zhenning Li:** validation, resources, data curation, writing – original draft. **Ruokun Wei:** validation, resources. **Huishuai Li** and **Zhenning Li:** resources, writing – review and editing. **Shaojun Li:** conceptualization, funding acquisition, supervision, writing – review and editing.

## Ethics Statement

All animal procedures performed in this study were performed strictly according to the international standards of animal care guidelines and have been approved by the Animal Care and Use Committee of Guangxi Medical University (No.: 202101031).

## Conflicts of Interest

The authors declare no conflicts of interest.

## Supporting information


**Data S1:** cns70566‐sup‐0001‐DataS1.pdf.

## Data Availability

All data generated or analyzed during this study are included in this published article.
