# Supplementary material for: The Role of P62/Nrf2/Keap1 Signaling Pathway in Lead‐Induced Neurological Dysfunction
Source: CNS Neurosci Ther. 2025 Sep 5;31(9):e70566. doi: 10.1111/cns.70566 (PMC12413483; doi:10.1111/cns.70566)
Supplement: Supplementary file 1 — Data S1: cns70566‐sup‐0001‐DataS1.pdf. [file CNS-31-e70566-s001.pdf]

We use the rainbow markers.

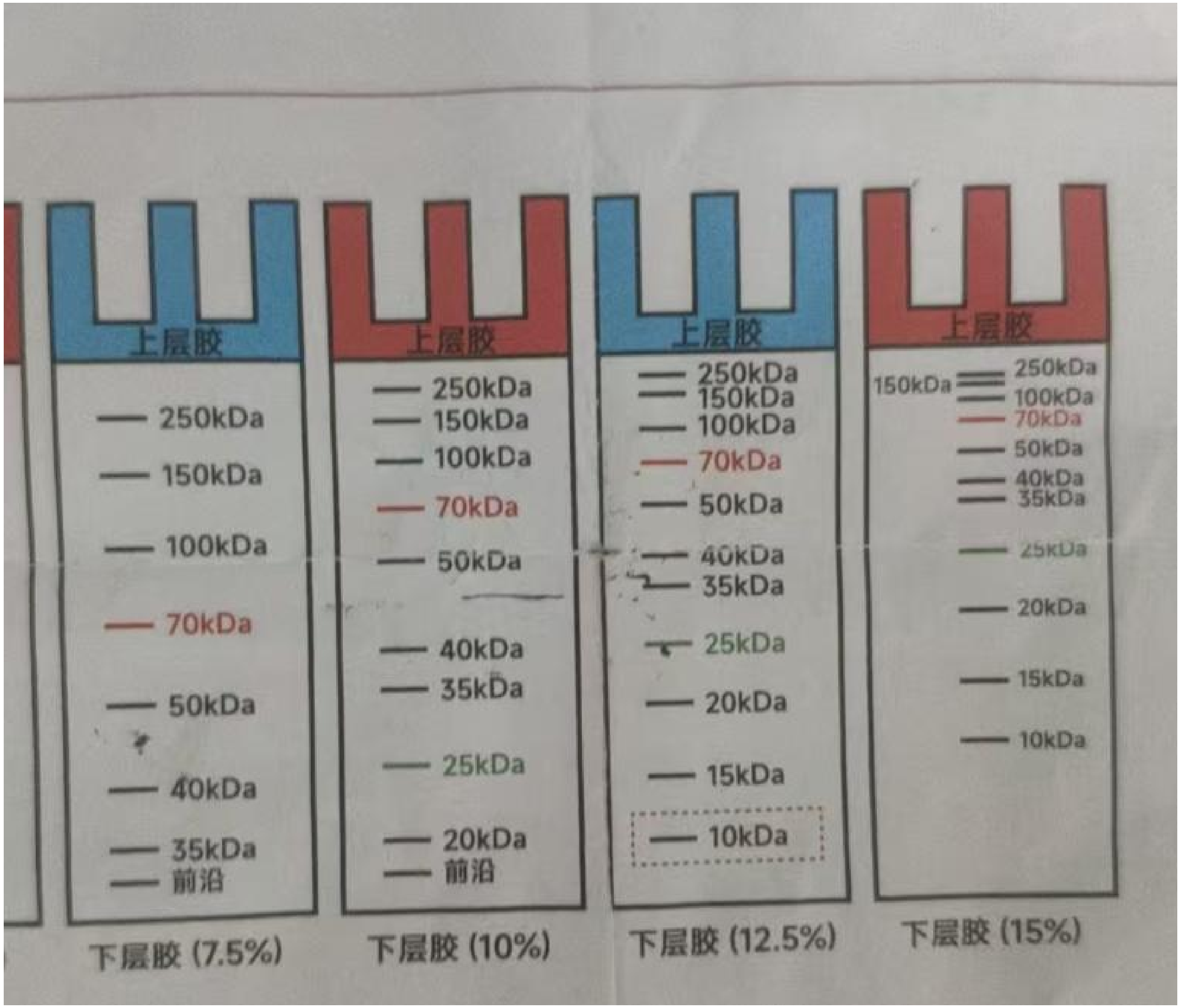

Figure 3B

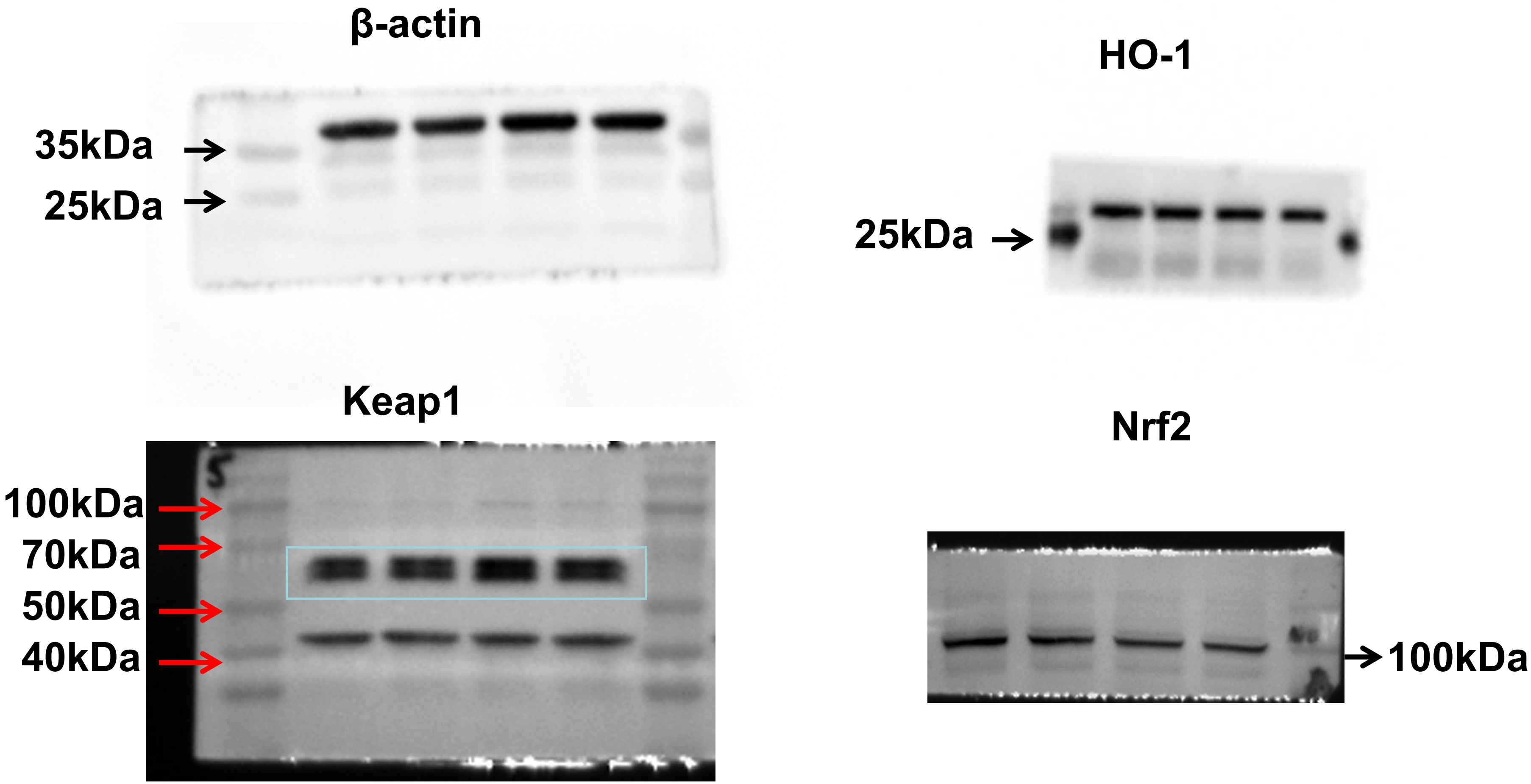
β-actin
35kDa
25kDa
HO-1
25kDa
Keap1
100kDa
70kDa
50kDa
40kDa
Nrf2
100kDa

Figure 3C

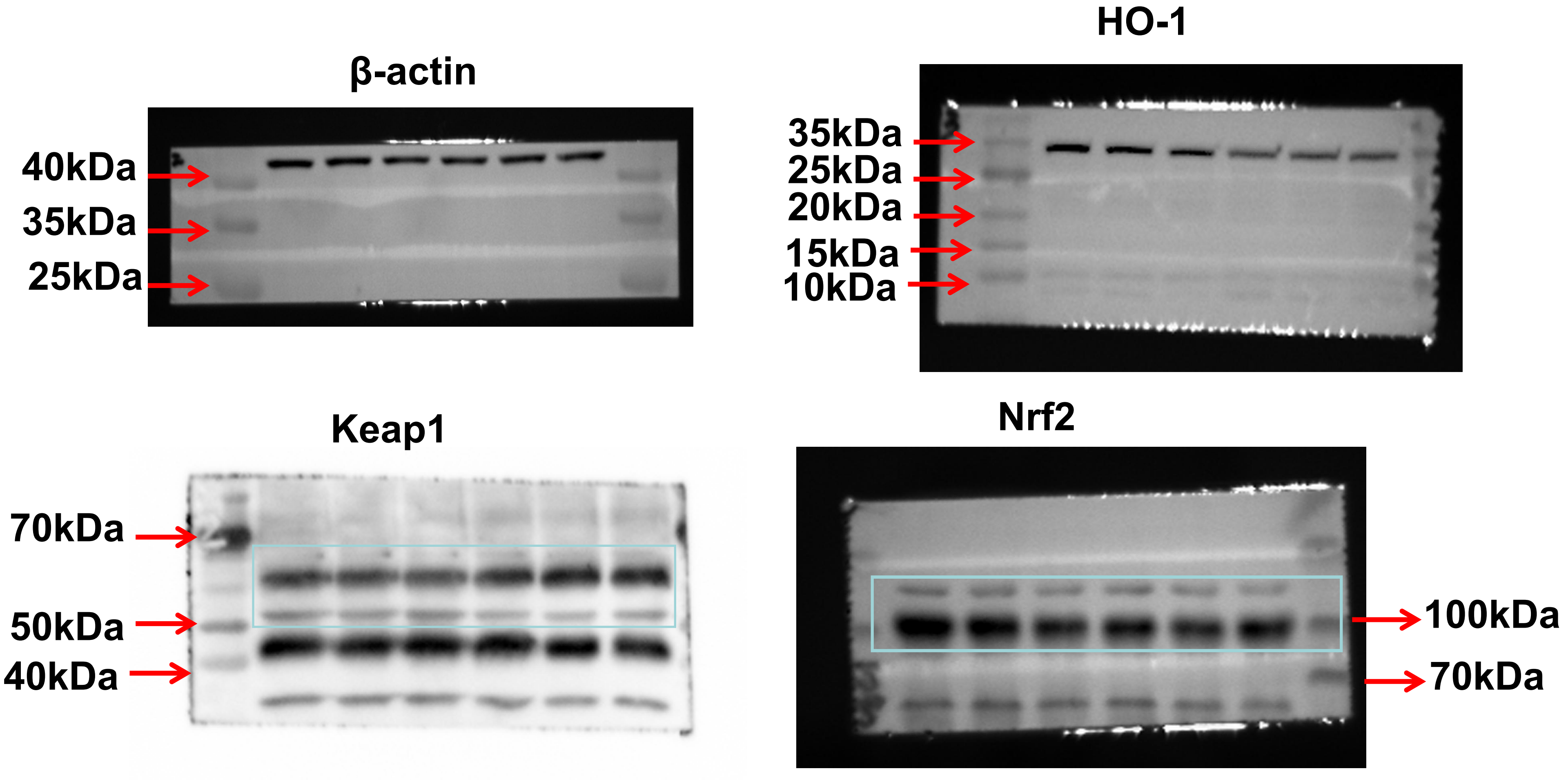

β-actin
40kDa
35kDa
25kDa
HO-1
35kDa
25kDa
20kDa
15kDa
10kDa
Keap1
70kDa
50kDa
40kDa
Nrf2
100kDa
70kDa

Figure 4A

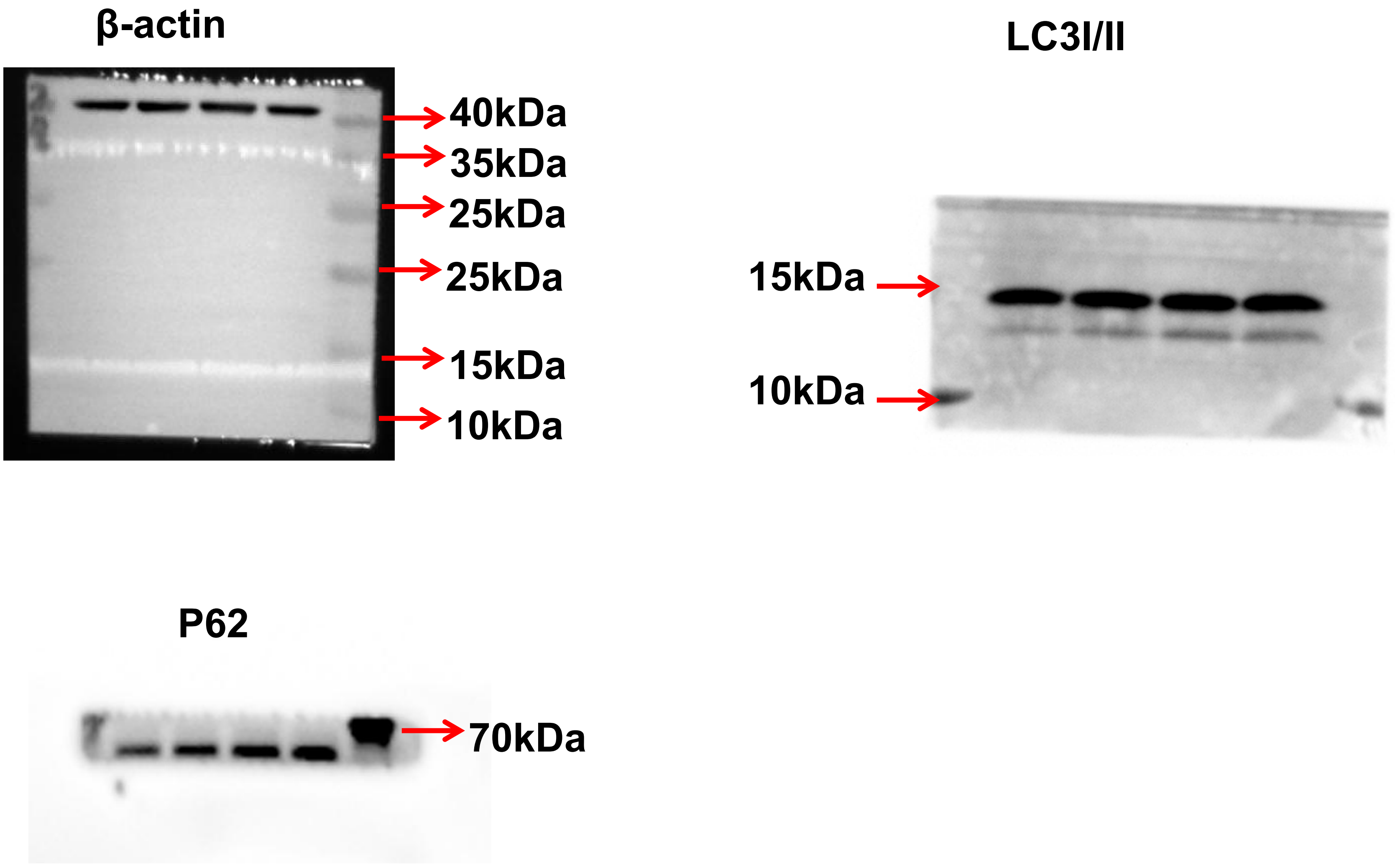

β-actin
40kDa
35kDa
25kDa
25kDa
15kDa
10kDa
LC3I/II
15kDa
10kDa
P62
70kDa

Figure 4C

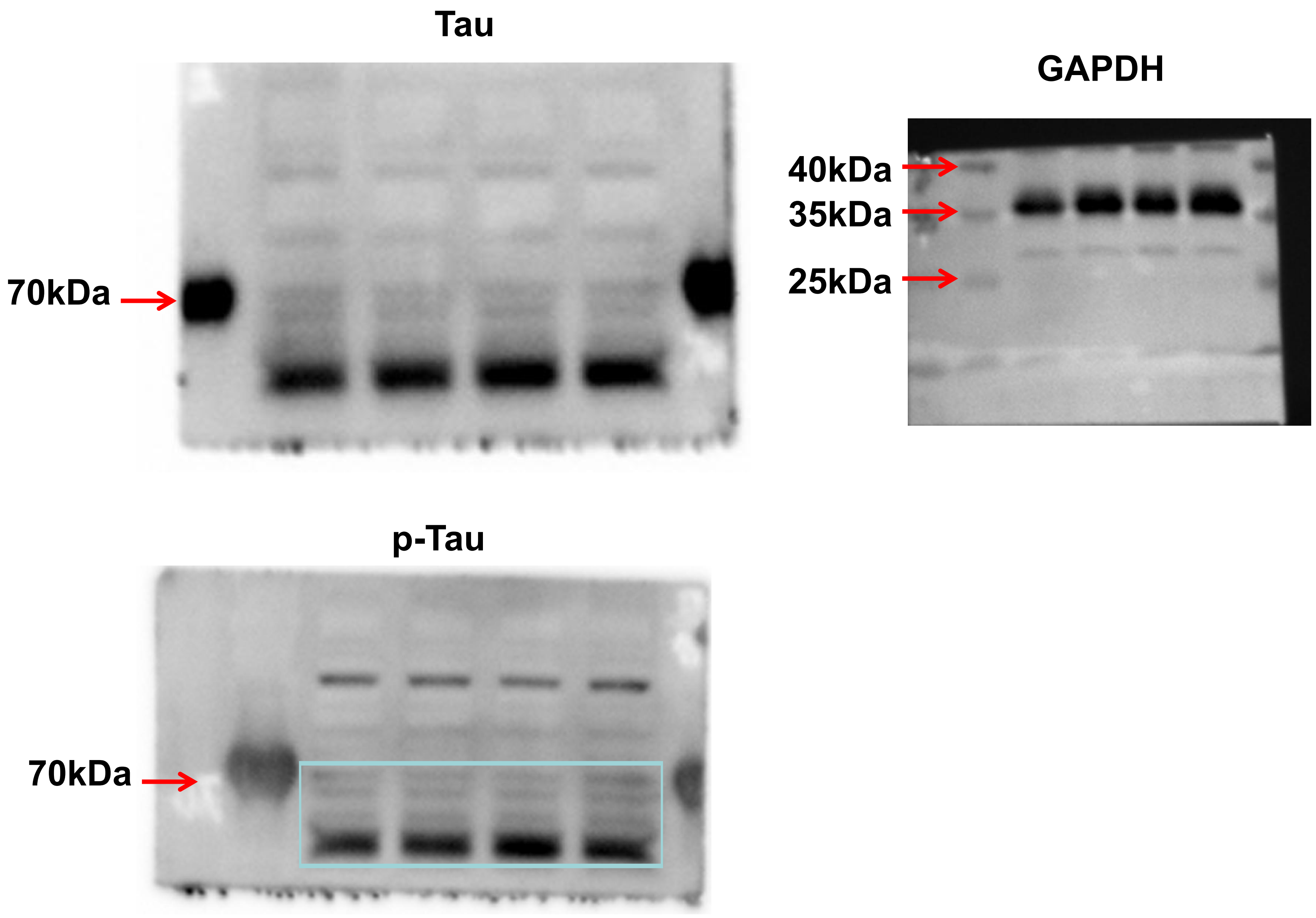

Figure 4D

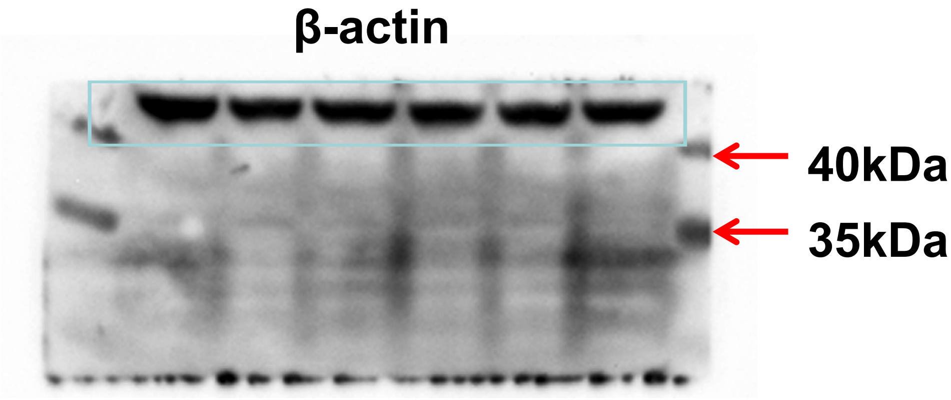

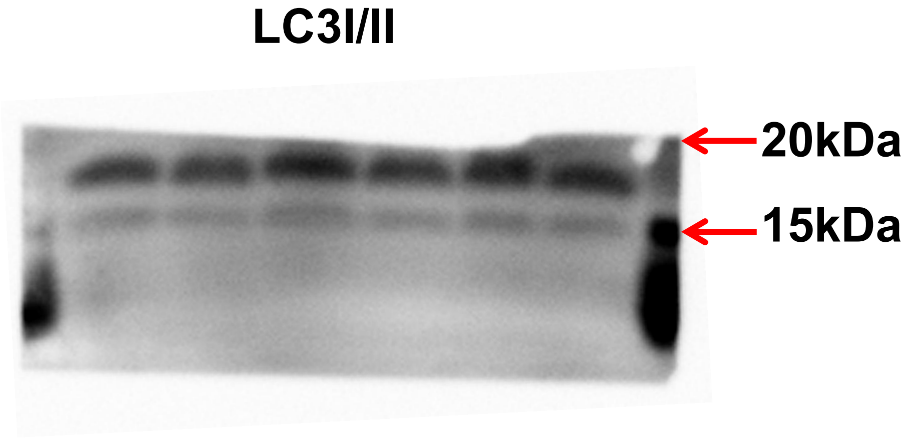

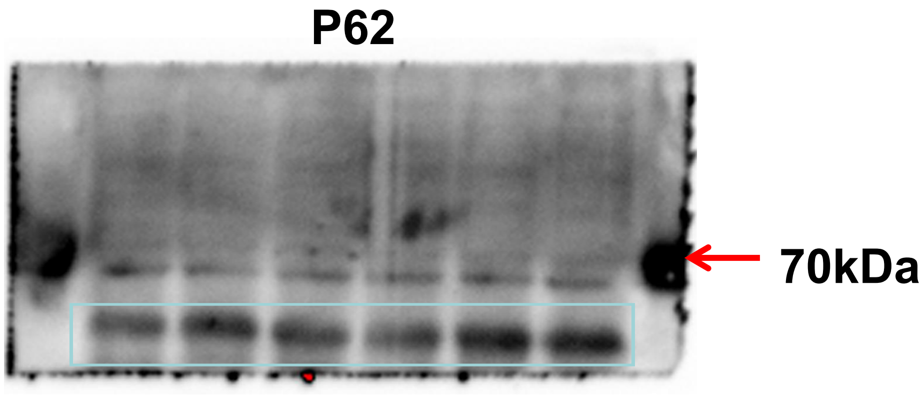

Figure 4E

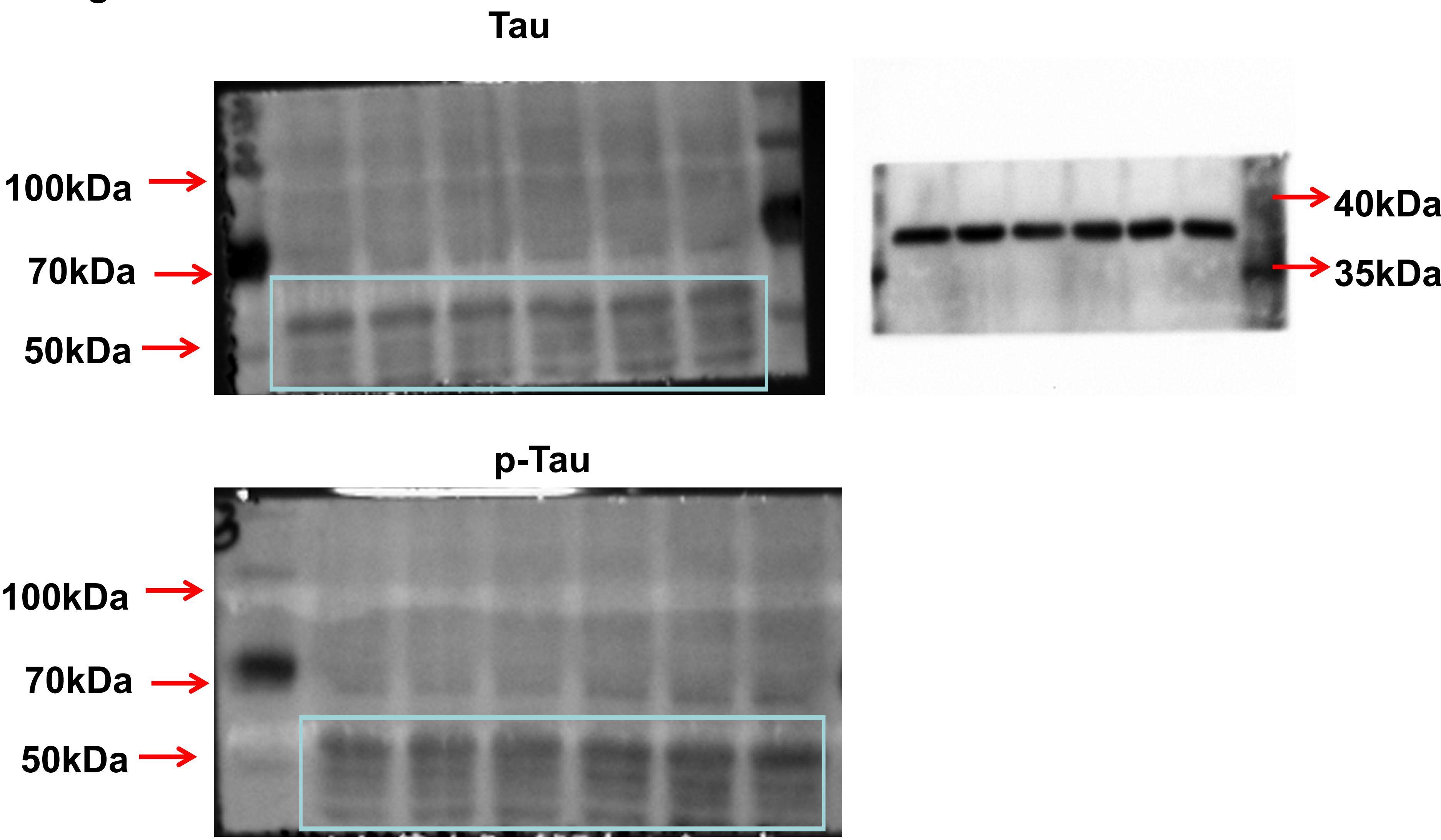

Figure 6B

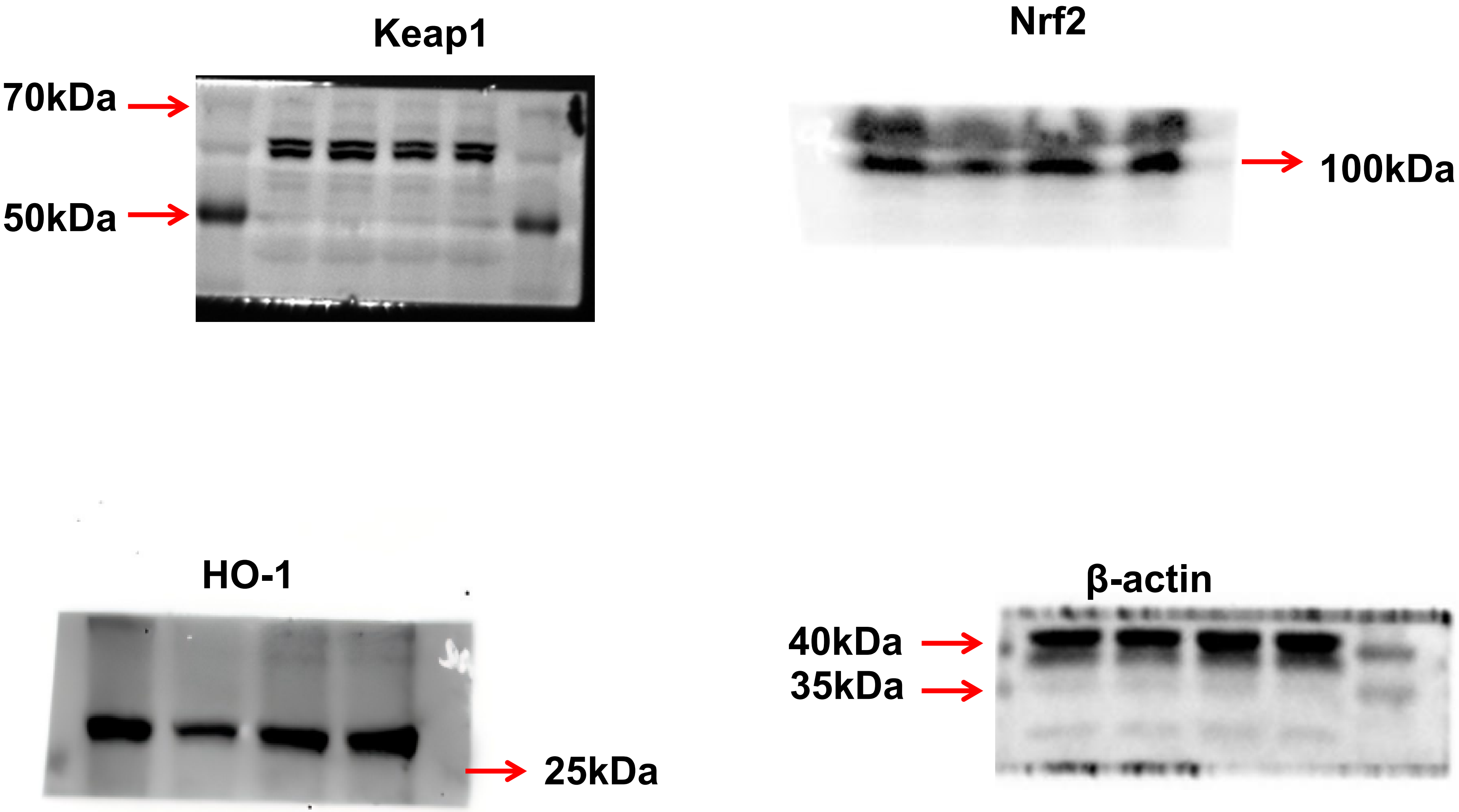

Figure 6C

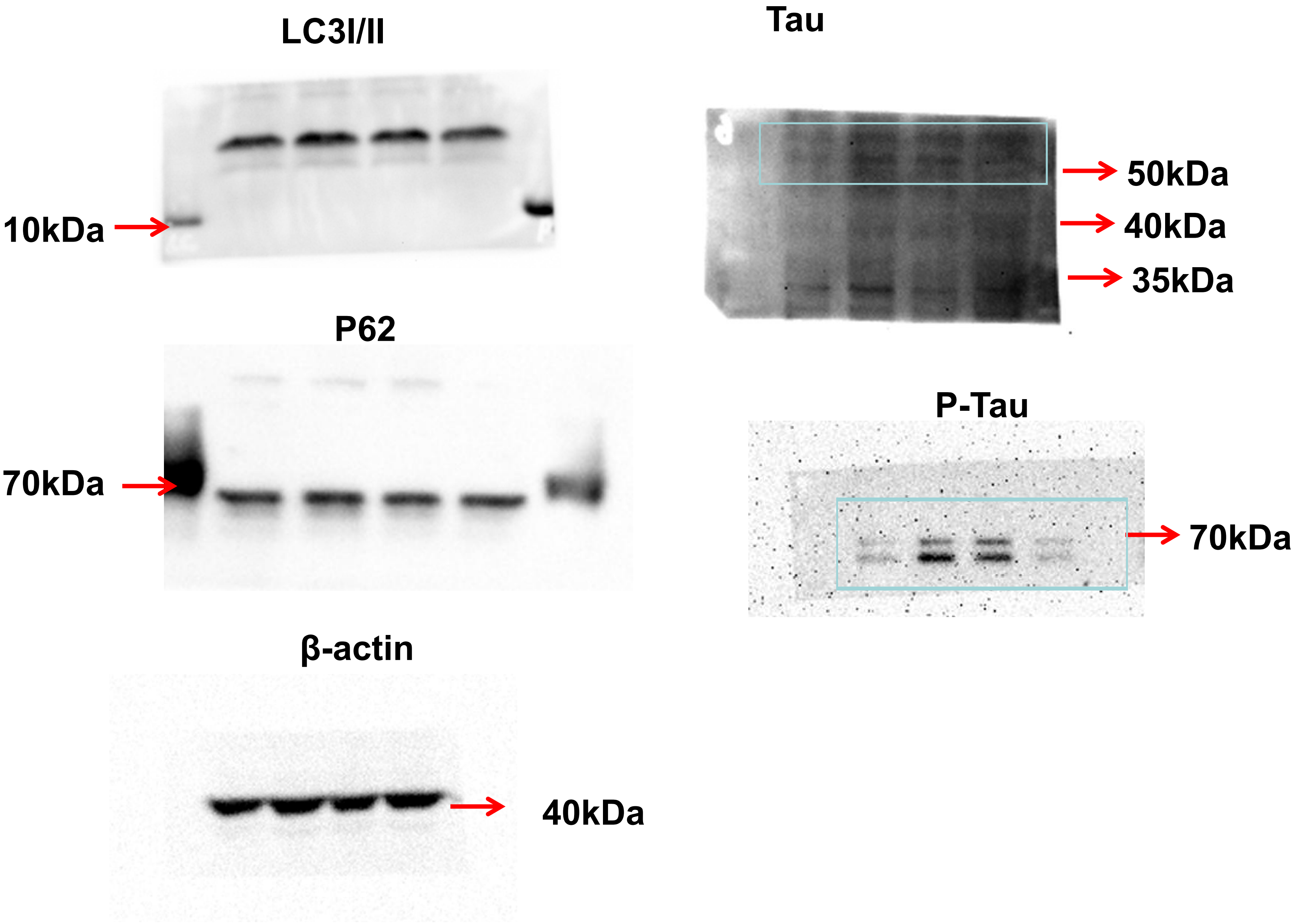
LC3I/II
10kDa
Tau
50kDa
40kDa
35kDa
P62
70kDa
P-Tau
70kDa
β-actin
40kDa

Figure 7A

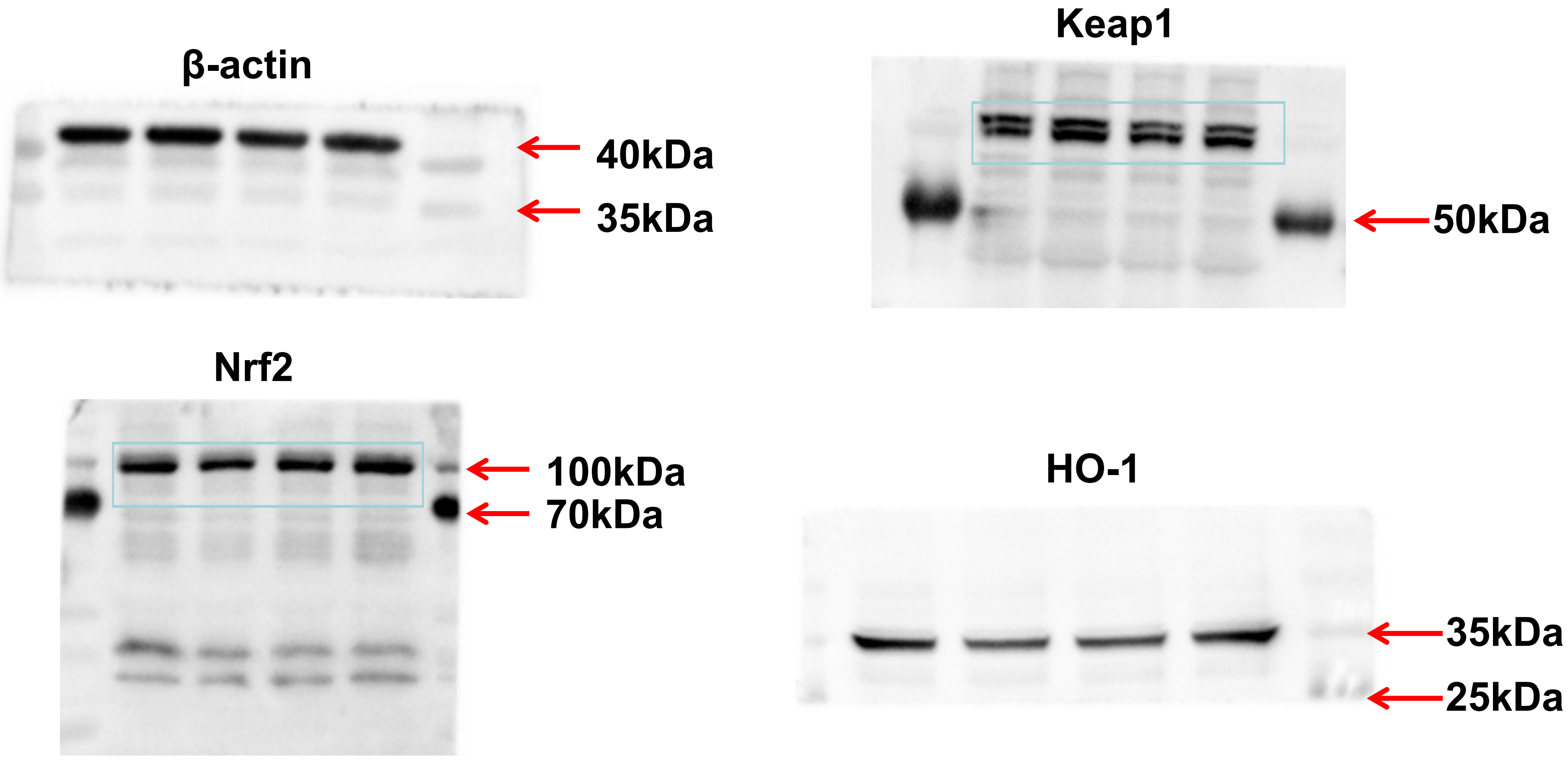
β-actin
40kDa
35kDa
Keap1
50kDa
Nrf2
100kDa
70kDa
HO-1
35kDa
25kDa

Figure 7B

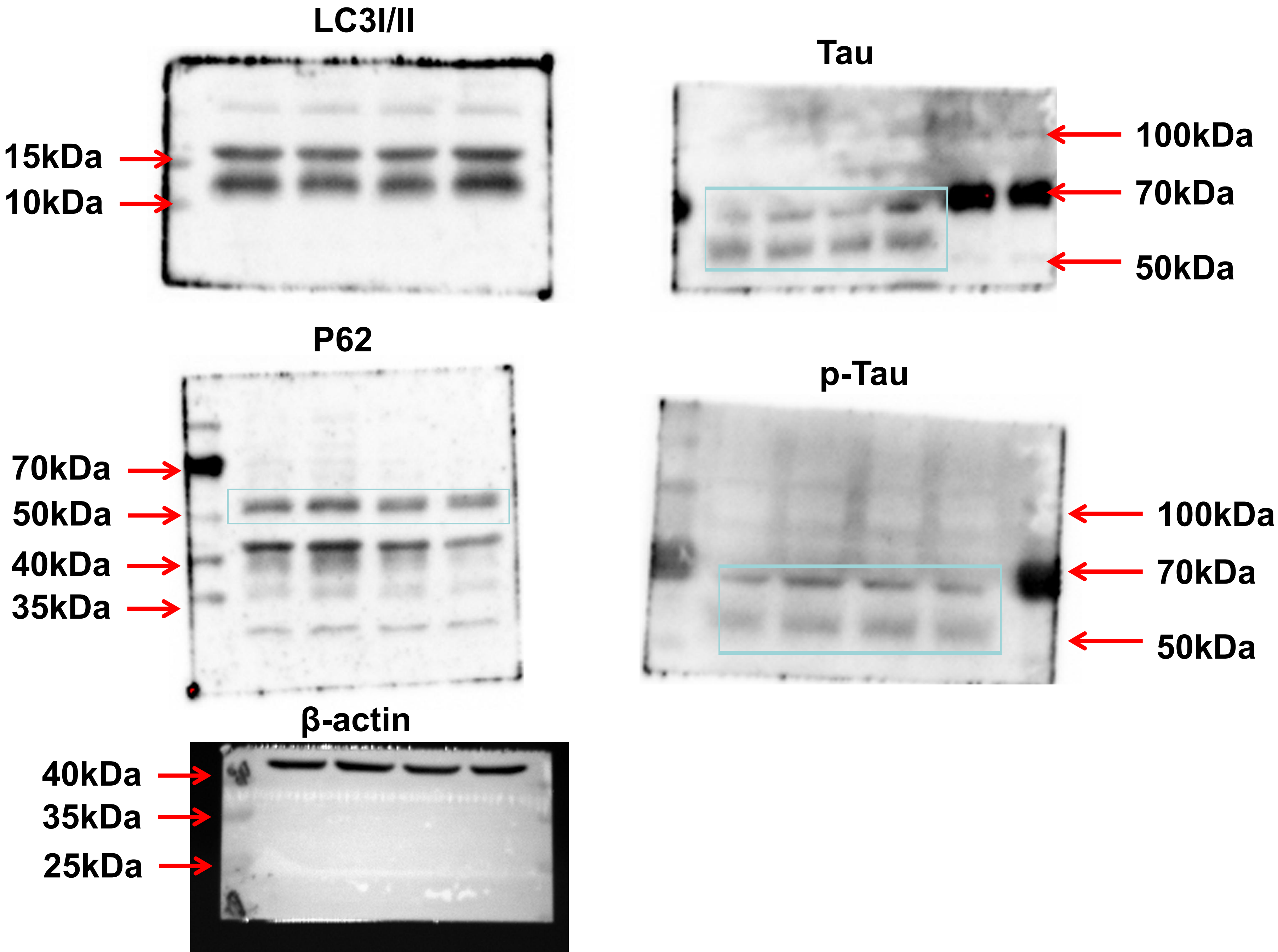
LC3I/II
15kDa
10kDa
Tau
100kDa
70kDa
50kDa
P62
70kDa
50kDa
40kDa
35kDa
p-Tau
100kDa
70kDa
50kDa
β-actin
40kDa
35kDa
25kDa

Figure 8A

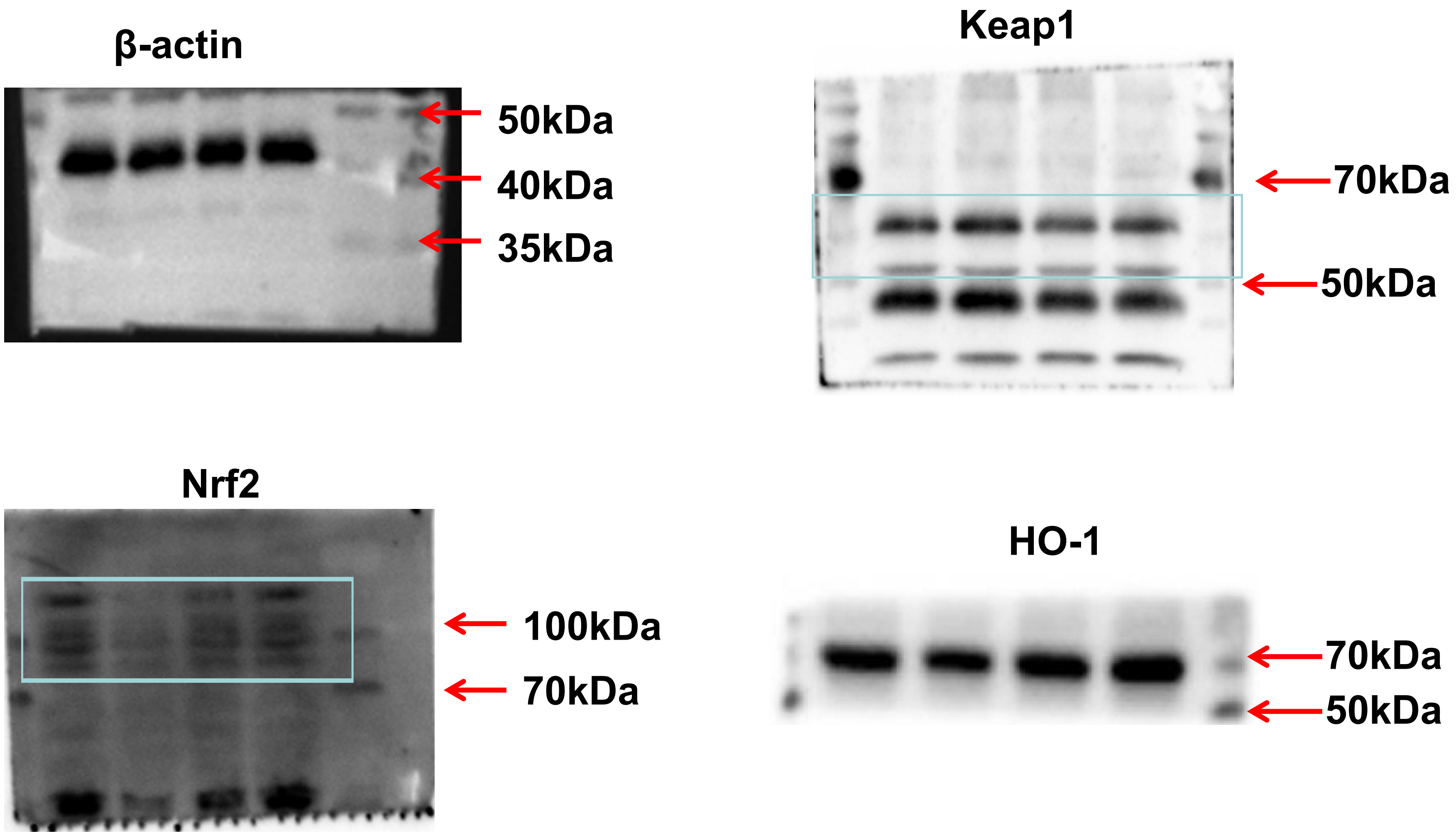
β-actin
50kDa
40kDa
35kDa
Keap1
70kDa
50kDa
Nrf2
100kDa
70kDa
HO-1
70kDa
50kDa

Figure 8B

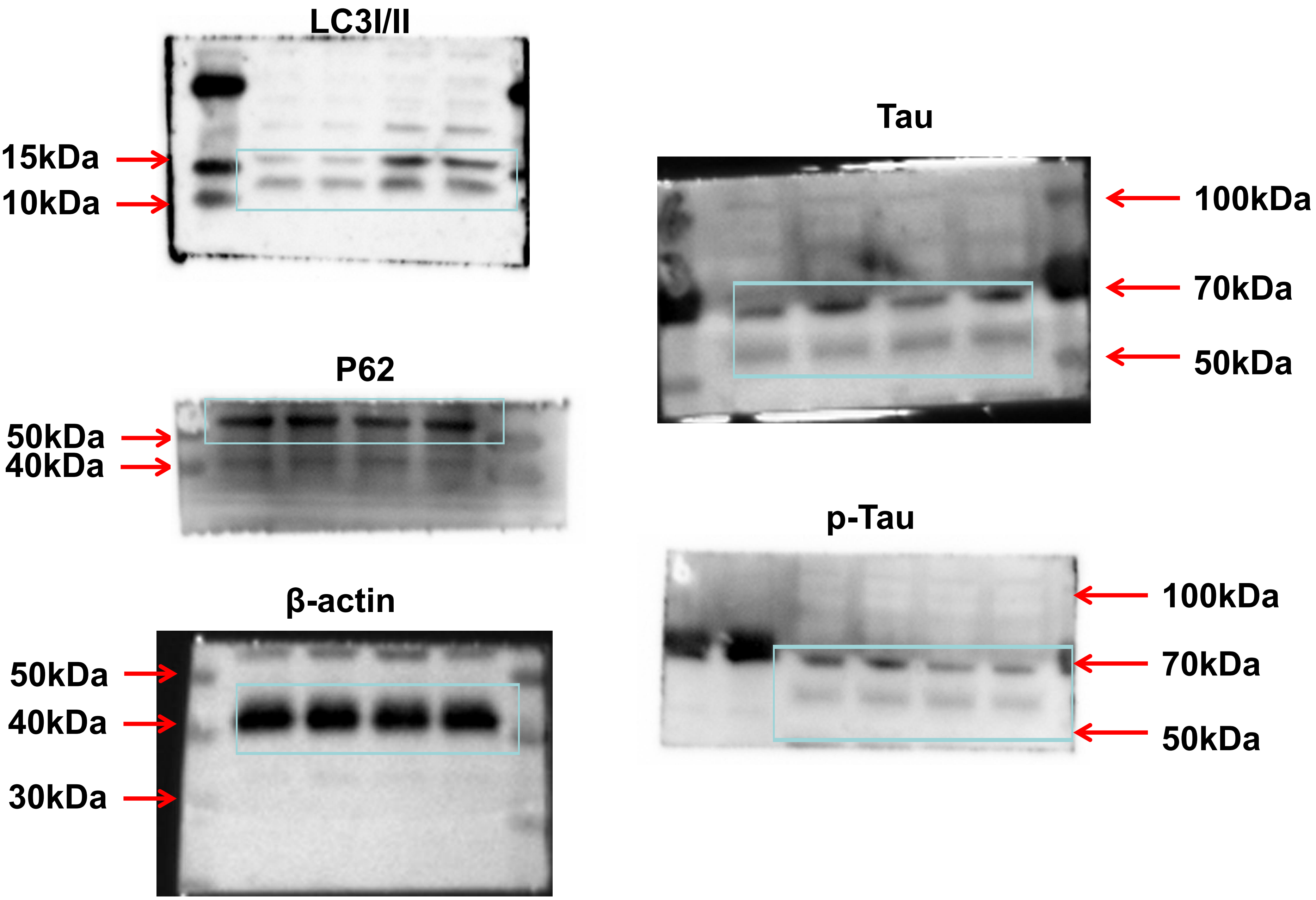
LC3I/II
15kDa
10kDa
P62
50kDa
40kDa
β-actin
50kDa
40kDa
30kDa
Tau
100kDa
70kDa
50kDa
p-Tau
100kDa
70kDa
50kDa
